# Thermal Management and Reliability Engineering of Advanced HBM Packages: Materials, Interfaces, and Integrated Design Strategies

**DOI:** 10.3390/mi17091065

**Published:** 2026-09-08

**Authors:** Hye Rin Do, Jun Ha Wee, Hwa Rim Lee, Young Chae Lee, Yunna Song, Sung Gyu Pyo

**Affiliations:** School of Integrative Engineering, Chung-Ang University, 84 Heukseok-ro, Dongjak-gu, Seoul 06974, Republic of Korea

**Keywords:** High Bandwidth Memory, thermal management, packaging reliability, interfacial thermal resistance, thermal interface material, underfill, hybrid bonding, thermal-reliability co-design

## Abstract

Advances in artificial intelligence, high-performance computing, and generative AI technologies have driven a rapid increase in the memory bandwidth and data throughput required of semiconductor systems, establishing High Bandwidth Memory (HBM)—which vertically stacks multiple DRAM dies—as a key enabling memory technology. However, increasing the stack count and shrinking the interconnect pitch in HBM not only intensify vertical heat accumulation and hotspot formation but also give rise to complex reliability issues, including thermo-mechanical stress arising from coefficient-of-thermal-expansion (CTE) mismatch, package warpage, interfacial delamination, Cu protrusion, void formation, and joint degradation. This review analyzes the heat-generation and heat-transfer mechanisms of HBM packages and examines package-level thermal management strategies based on thermal interface materials, underfill, non-conductive film, epoxy molding compound, heat spreaders, and high-thermal-conductivity composites. It further summarizes the current crowding, electromigration, Cu–dielectric interfacial defects, and thermo-mechanical failure mechanisms that arise at fine-pitch interconnects and hybrid-bonding interfaces, together with the material and process design strategies developed to mitigate them. In addition, structure-based thermal management technologies—thermal TSVs, embedded cooling, and hybrid bonding—are compared. This review emphasizes that the thermal bottlenecks and reliability degradation of HBM are interconnected through interfacial thermal resistance, interfacial adhesion, residual stress, and interfacial defects, and proposes that next-generation, highly stacked HBM requires a multi-scale thermal-reliability co-design that integrally controls the heat-, stress-, and current-transfer pathways across the entire package and interconnect domain, rather than relying on the improvement of individual material properties alone.

## 1. Introduction

Advances in artificial intelligence, high-performance computing, and generative AI technologies have driven a rapid increase in the data throughput and memory bandwidth required of semiconductor systems [1,2,3]. However, the growth rate of memory bandwidth remains comparatively limited relative to the improvement in processor computational performance, intensifying the memory-wall problem in which data-movement latency and power consumption constrain overall system performance. High Bandwidth Memory (HBM) has attracted attention as a key technology for overcoming this limitation [4,5].

HBM achieves high bandwidth and superior power efficiency by vertically stacking multiple DRAM dies and connecting them at high density through through-silicon vias (TSVs) [6,7,8,9]. Because it can also be integrated within the same package as a GPU or AI accelerator, HBM has become a core memory architecture for next-generation AI and HPC systems [10,11,12]. However, increasing the number of stacked dies and shrinking the interconnect pitch lengthen the heat-transfer path, accumulate interfacial thermal resistance, and raise power density, thereby intensifying internal heat accumulation and hotspot formation [13,14,15].

HBM packages are composed of materials with markedly different thermal and mechanical properties, including Si, Cu, underfill, non-conductive film, epoxy molding compound, and substrate [16,17,18,19]. When temperature changes occur, the resulting mismatch in coefficient of thermal expansion (CTE) among these materials generates thermo-mechanical stress, which can lead to warpage, delamination, Cu protrusion, and interconnect failure. Furthermore, once interfacial delamination or voids form, the local thermal resistance increases, which in turn raises the local temperature and stress, driving a coupled degradation process [20,21,22].

Conventional micro-bump structures, meanwhile, become increasingly vulnerable to current crowding, electromigration, void formation, and degraded joint reliability as the pitch decreases [1,7,23,24]. To overcome this limitation, hybrid bonding—which simultaneously forms Cu–Cu direct bonds and dielectric–dielectric bonds—has been proposed as a next-generation HBM interconnect technology [25,26,27,28]. Hybrid bonding can achieve ultra-fine pitch and low electrical resistance, but it is highly sensitive to surface roughness, native oxide, contamination, flatness, and interfacial voids, and therefore requires precise interfacial control [5,29,30].

To address these issues, packaging materials such as thermal interface materials, high-thermal-conductivity underfills, non-conductive films, epoxy molding compounds, and carbon-based composites are being developed, alongside structure-based thermal management technologies such as thermal TSVs, heat spreaders, and embedded cooling [9,31,32,33,34,35,36,37]. However, high filler loading intended to improve thermal conductivity also increases viscosity and elastic modulus, which can degrade gap-filling capability and stress-relaxation performance; consequently, improving a single material property alone is insufficient to simultaneously resolve HBM’s thermal management and reliability challenges [2,38,39,40,41,42,43,44].

Therefore, next-generation HBM packaging must integrally consider not only material thermal conductivity but also interfacial thermal resistance, adhesion strength, CTE, residual stress, current transport, and processability [10,18,45]. This review analyzes the heat-transfer mechanisms and major thermal and mechanical reliability issues of HBM packages, and comprehensively examines package-level thermal management materials and interconnect-level interfacial design technologies. It further compares hybrid bonding with structure-based cooling technologies and proposes directions for multi-scale thermal-reliability co-design to ensure the long-term reliability of highly stacked HBM.

## 2. Reliability Challenges in HBM Packages

### 2.1. HBM Architecture and Heat Generation

HBM has a three-dimensional integration structure in which multiple DRAM dies are vertically stacked, with each die connected to the base die through TSVs and micro-bumps or a hybrid-bonding structure [1,5,23,46,47]. The stacked HBM is connected to a GPU, CPU, or AI accelerator through a silicon interposer or an advanced substrate [3,4,12]. This structure shortens the data-transfer distance and increases I/O density, but it also concentrates numerous heat sources within a small area.

Heat generation in HBM originates mainly from the memory banks and peripheral circuits of the DRAM dies, as well as from the base or logic die at the bottom of the stack. In particular, relatively high power density can develop in the PHY and logic regions responsible for high-speed data input/output [6,9,11]. When HBM and a GPU are placed on the same interposer, the overall temperature distribution is affected not only by the HBM’s own heat generation but also by heat transferred from the adjacent GPU.

As the number of stacked HBM dies increases, not only does the total heat generation increase, but the length of the heat-transfer path from the internal dies to the external cooling surface also grows. Heat generated in dies located near the center of the stack or far from the cooling surface must pass through multiple silicon layers and bonding interfaces, making it difficult to dissipate efficiently [7,8,10,13]. As a result, the vertical temperature gradient increases with stack count, raising the likelihood of localized hotspot formation at specific locations.

The internal temperature of HBM is determined not only by the amount of heat generated but also by the position of the heat spreader, the cooling boundary condition, die thickness, the thermal conductivity of the bonding layer, and TSV placement [14,48,49,50]. Consequently, HBM devices with the same total power consumption can exhibit different peak temperatures and temperature distributions depending on the stacking structure and heat-transfer path. In particular, when the heat source and the cooling surface are positioned asymmetrically, the internal temperature distribution of the stack likewise becomes asymmetric.

**Figure 1:**
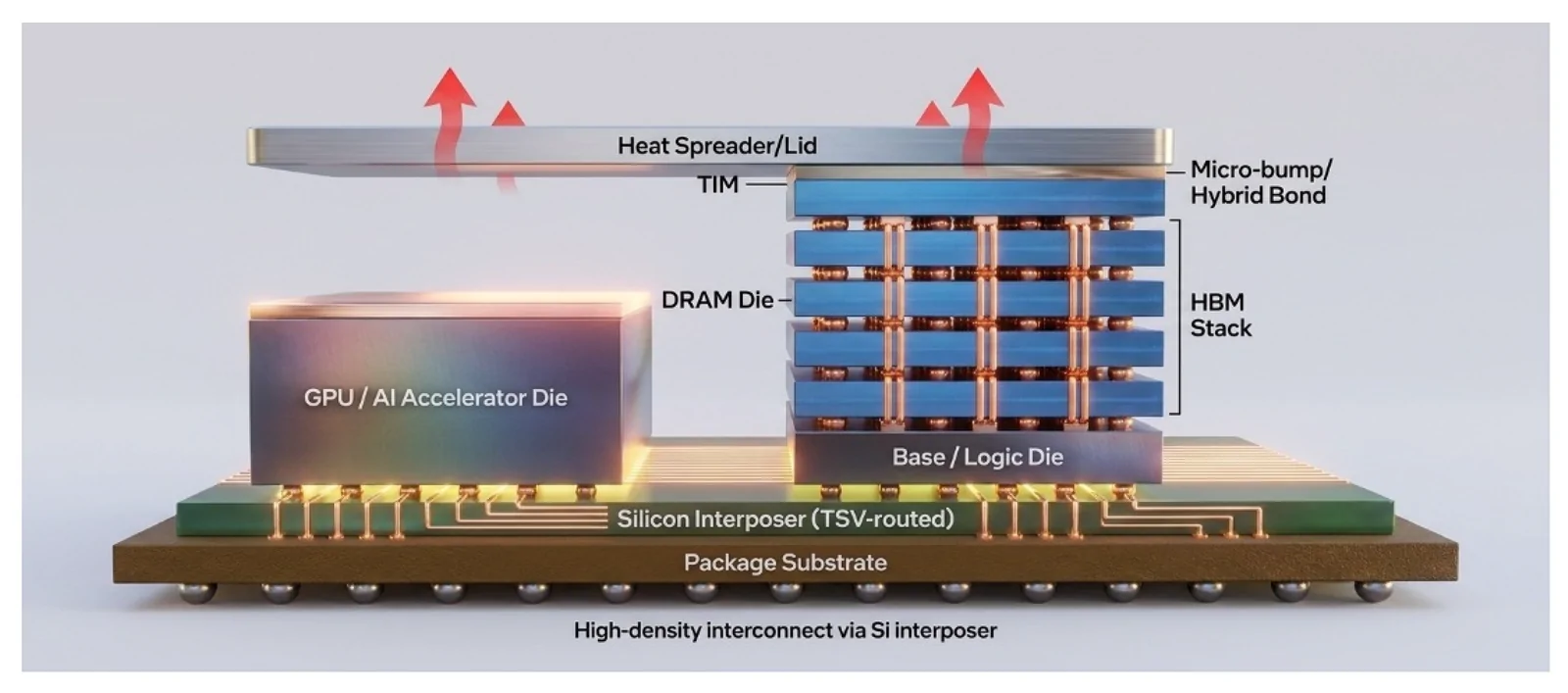
Schematic of HBM packaging architecture [21]. *Original schematic prepared by the authors based on the packaging architecture reported in Ref.* [21].

The overall packaging architecture, including the GPU/accelerator die, the HBM stack, the silicon interposer, and the heat spreader, is illustrated in Figure 1.

### 2.2. Heat Transfer Path and Interfacial Thermal Resistance

**Figure 2:**
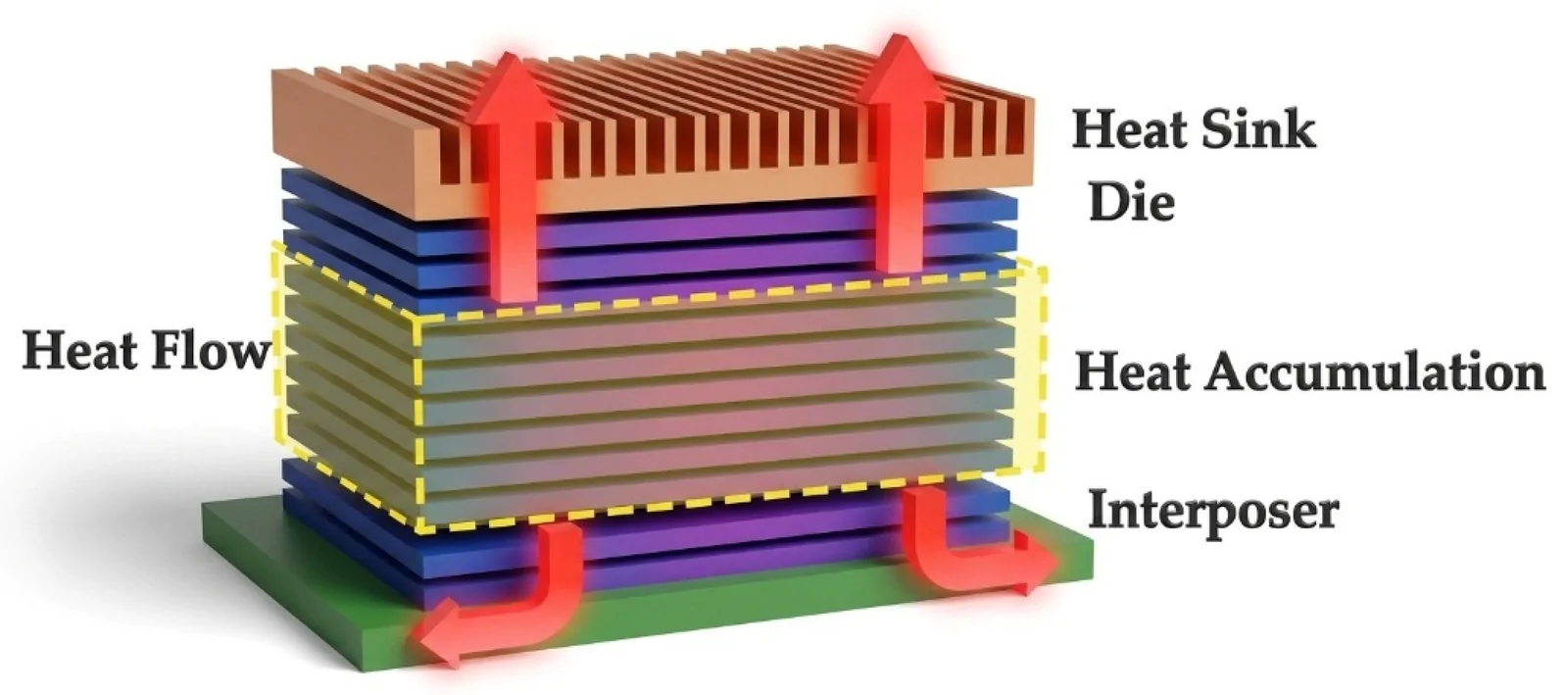
Heat dissipation pathways and thermal accumulation in a 3D-stacked memory structure [7]. *Reproduced from Ref.* [7], *an open-access article published in Electronics* (*MDPI*) *under the terms of the Creative Commons Attribution* (*CC BY*) *license.*

Heat generated in HBM is dissipated to the outside through the silicon die, TSVs, micro-bumps, bonding layers, underfill or NCF, TIM, and the heat spreader [31,34,39,51]. The thermal resistance of each material layer is determined by its thickness and thermal conductivity, but in an actual package, the interfacial thermal resistance that arises at the contact interface between dissimilar materials has a substantial effect on overall heat-transfer performance. Figure 2 illustrates representative heat dissipation pathways and the resulting thermal accumulation in such a 3D-stacked memory structure.

Silicon and Cu have relatively high thermal conductivity, whereas the underfill, NCF, and dielectric positioned between dies have comparatively low thermal conductivity. In particular, in highly stacked HBM structures where inter-die bonding layers are repeated many times, even a small thermal resistance at each individual interface can accumulate to a substantial effect across the entire stack [40,44,52]. Accordingly, the vertical heat-transfer performance of HBM can be limited more by the characteristics of the repeated bonding layers and interfaces than by the thermal conductivity of any single material.

Interfacial thermal resistance is influenced by surface roughness, actual contact area, interfacial voids, contamination, and the phonon-transport characteristics between the two materials [32,36,53]. Even when a bonded surface appears flat to the eye, the actual contact area can be smaller than the total area because of microscale surface asperities. Air or microvoids exist in the non-contacting regions and act as localized thermal barriers that impede heat transfer.

TSVs and Cu interconnects can serve as low-thermal-resistance vertical heat-transfer paths [54,55,56]. However, because TSVs in HBM are primarily placed to carry electrical signals and power, it is difficult to optimize their position and density based on heat transfer alone [31,55]. In addition, the dielectric liner and barrier layer surrounding the TSV can limit direct heat transfer between the Cu and the silicon. Therefore, the thermal effect of TSVs should be evaluated not only in terms of the high thermal conductivity of the metal but also in terms of TSV density, diameter, placement, and the surrounding interfacial structure.

### 2.3. Thermo-Mechanical Stress and Package Deformation

HBM packages are composed of a variety of materials—Si, Cu, solder, polymer, and package substrate—whose CTE and elastic modulus differ substantially from one another. In general, Si has a lower CTE than Cu and polymer-based packaging materials, so when temperature changes during fabrication or operation, each material undergoes thermal deformation of a different magnitude [16,17,19,57,58]. However, because the free expansion and contraction of individual materials in a stacked structure is constrained by adjacent layers, thermo-mechanical stress can become concentrated within the materials and at interfaces, particularly at die corners and structural discontinuities [16,17,19,59]. The magnitude and distribution of this stress are determined not only by the CTE mismatch between materials but also, in combination, by the elastic modulus, layer thickness, structural constraint conditions, the stress-free temperature, and the overall thermal history of the process [18,20].

**Figure 3:**
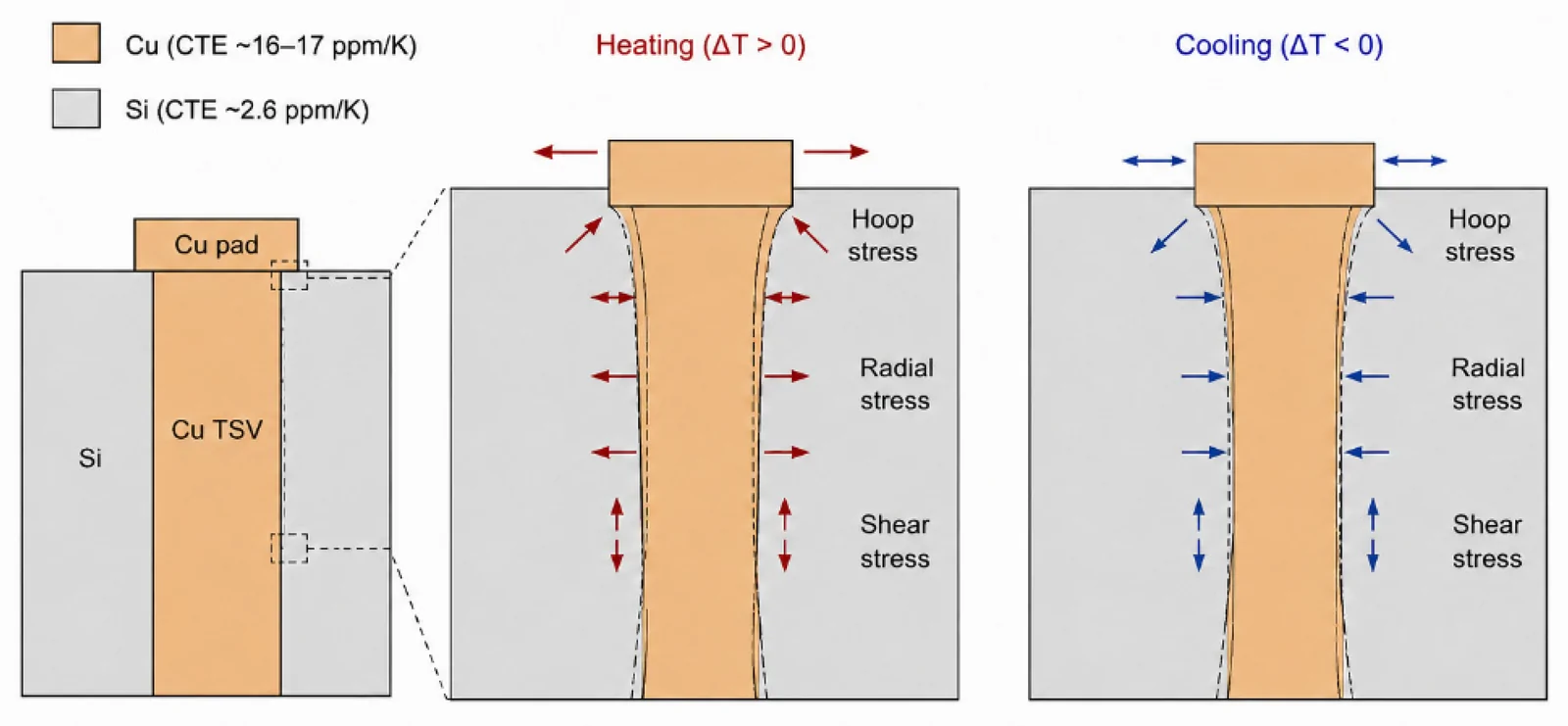
Thermo-mechanical stress evolution around a Cu TSV during thermal cycling. *Original schematic prepared by the authors.*

As can be inferred from the coefficients of thermal expansion (CTE) of Si and Cu shown in Figure 3, the Cu within a TSV tends to expand to a greater degree than the surrounding silicon. As temperature rises, the expansion of Cu is constrained by the silicon, which can generate radial, hoop, and shear stresses at the TSV sidewall and the surrounding dielectric [16,50,57]. This stress can affect the electrical characteristics of the region between the TSV and the transistor area, and under repeated thermal history it can progress into interfacial cracking and delamination.

Cu protrusion is a phenomenon in which the top of a Cu pad or TSV protrudes above the surrounding surface, arising from the CTE mismatch between Cu and the surrounding Si or dielectric together with the relaxation of residual stress generated by plastic deformation, high-temperature creep, grain growth, and the electroplating process of Cu [21,60,61]. Non-uniform Cu protrusion degrades the coplanarity of the bonding interface and concentrates contact pressure at specific pads. Because hybrid bonding requires simultaneous contact between Cu and dielectric surfaces, even a height difference on the order of several to several tens of nanometers can affect bonding quality.

At the package level, warpage can arise from differences in CTE and elastic modulus among the various material layers [20,22,62,63]. Cure shrinkage and residual stress generated during the curing of underfill or EMC also affect package deformation. In highly stacked HBM, deformation accumulated from each die and bonding layer makes it increasingly difficult to control overall package curvature and die-level warpage as the stack count increases.

Warpage is not merely a change in shape; it also affects bonding alignment, underfill flow, and interconnect contact condition. Non-uniform deformation concentrates tensile or shear stress at certain interconnects, which can lead to cracking, open failure, and delamination [17,18,19]. Conversely, once interfacial delamination occurs, local stiffness decreases and deformation becomes further concentrated, which can intensify warpage even further.

### 2.4. Fine-Pitch Interconnect and Hybrid Bonding Reliability

**Figure 4:**
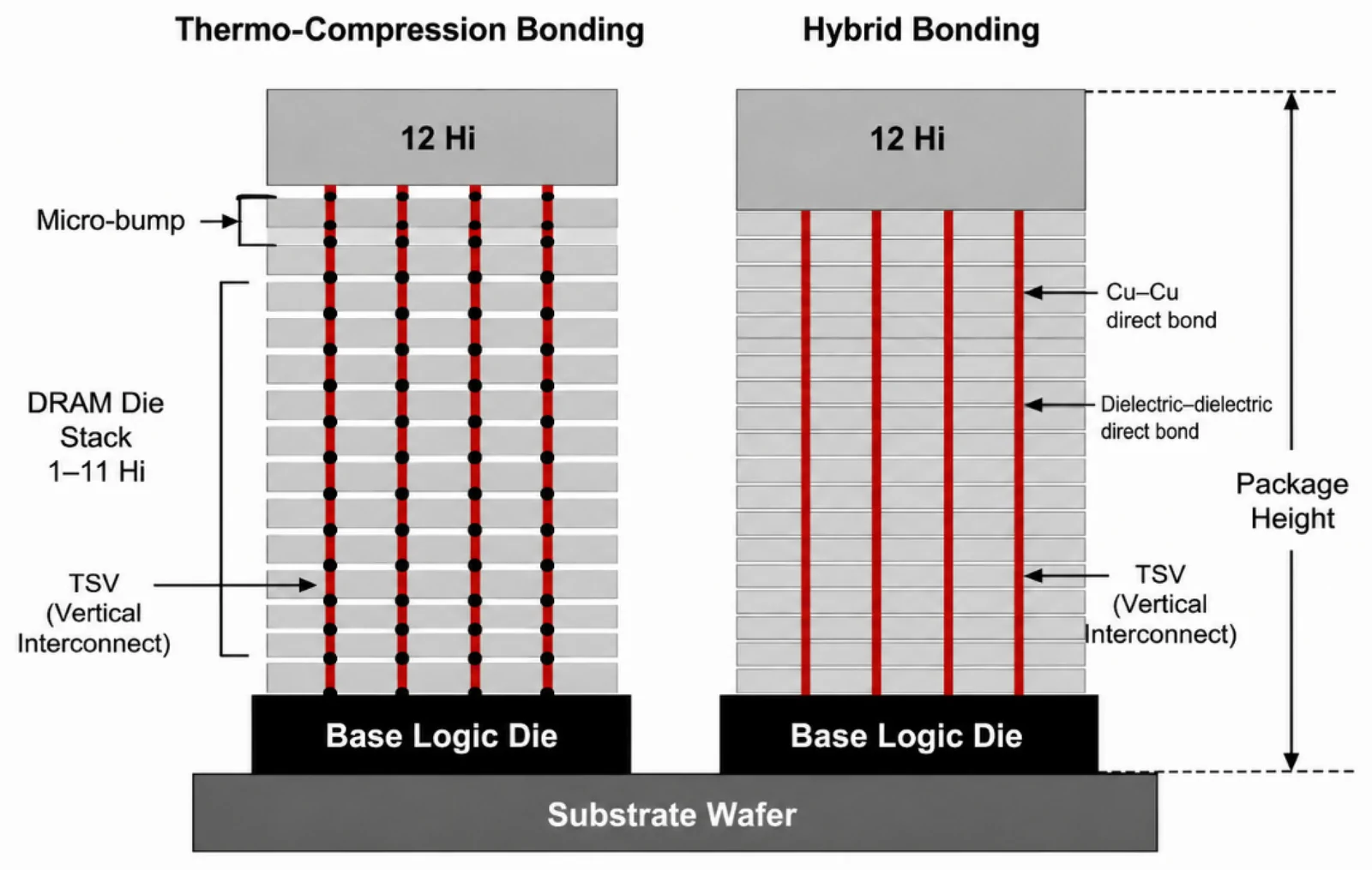
Comparison of micro-bump and hybrid bonding in HBM. *Original schematic prepared by the authors.*

Conventional HBM has widely relied on solder-based micro-bumps to connect stacked dies [64,65,66,67,68]. However, as the interconnect pitch decreases, the diameter and height of the bumps decrease correspondingly, and the alignment precision and shape uniformity required during the bonding process increase substantially. At narrow spacings, underfill or NCF can have difficulty fully filling the gaps between bumps, resulting in voids and unfilled regions. Figure 4 compares the micro-bump and hybrid-bonding interconnect structures.

In fine-pitch micro-bumps, current crowding is likely to occur near the entrance where the wiring connects to the bump and near the UBM edge, where the current path narrows abruptly [24,59,66,67]. The resulting local increase in current density promotes Joule heating and electromigration; as electrons flow, metal atoms migrate, typically causing atomic depletion and void formation on the cathode side and metal accumulation with intermetallic-compound growth on the anode side. In solder-based joints, Kirkendall voids can also form due to the asymmetric diffusion of Cu and Sn and the growth of intermetallic compounds. This degradation reduces the effective cross-sectional area of the interconnect and can lead to increased contact resistance, local heating, and eventual open failure.

Hybrid bonding is a technology that eliminates solder bumps and forms Cu–Cu bonding and dielectric–dielectric bonding at the same interface [5,7,29,30,56]. This approach can substantially reduce interconnect pitch and is advantageous for reducing the electrical resistance as well as the parasitic resistance and capacitance of the joint. Because it eliminates some of the interfaces previously occupied by solder and underfill and forms a direct Cu–Cu connection, it can also reduce vertical thermal resistance when sufficient contact area and a low void fraction are secured. For these reasons, hybrid bonding is regarded as a core interconnect technology for next-generation HBM and highly integrated 3D packaging.

However, the bonding quality and long-term reliability of hybrid bonding are highly sensitive to surface condition. Native oxide and organic contamination on the Cu surface impede Cu atomic diffusion and metallic bonding, and the influence of these surface defects becomes greater as the bonding temperature decreases [69,70,71,72,73]. At the dielectric interface, surface hydroxylation, surface energy, and moisture condition affect the initial pre-bonding strength and subsequent annealing behavior [26,27,74]. Surface particles can also form localized gaps that block the surrounding contact, causing interfacial voids far larger than the particle size itself. In fine-pitch structures, a defect of the same size occupies a larger fraction of the total pad area, so its electrical and thermal impact becomes correspondingly greater.

Cu dishing, dielectric erosion, and pad-height variation arising from the CMP process also limit bonding quality [21,28,75,76]. If the Cu is excessively recessed, initial contact may not form; excessive protrusion, on the other hand, can cause localized pressure concentration and dielectric damage. Hybrid bonding therefore requires the simultaneous, precise control of the surface flatness, roughness, and height difference of both the Cu and the dielectric.

### 2.5. Coupled Failure Propagation

The thermal, mechanical, and electrical degradation processes that occur in HBM do not proceed independently; rather, they develop in a mutually coupled manner in which one degradation phenomenon promotes another damage mechanism. A local hotspot does not so much change the CTE mismatch between materials itself as it amplifies the local temperature rise and temperature gradient, thereby increasing the thermal-deformation mismatch and thermo-mechanical stress around the TSV and bonding interface. Repeated application of this stress can lead to interfacial cracking and delamination; in the delaminated region, the actual contact area decreases and a low-thermal-conductivity gap or void forms, increasing the interfacial thermal resistance [16,18,22,39]. As a result, the temperature in adjacent regions rises further, and a positive-feedback behavior can emerge in which thermal stress and crack propagation continue to accelerate.

Interconnect voids directly link electrical and thermal degradation because they simultaneously reduce the current path and the heat-transfer area. When the effective conductive cross-section decreases due to void formation, current becomes concentrated in the remaining region, increasing the local current density and Joule heating [24,59,66,77]. The elevated temperature promotes metal-atom diffusion and electromigration, causing further void growth and an increase in interconnect resistance. Because the increased resistance in turn raises local heat generation, a repeating degradation cycle can form in which void growth, current crowding, and Joule heating mutually amplify one another. At hybrid-bonding interfaces as well, initial microscale defects such as Cu non-contact, particle-induced voids, and non-uniform Cu recess or protrusion can simultaneously increase contact resistance, local heat generation, and interfacial stress concentration, and under repeated thermal and electrical loading these defects can progress into cracking and delamination.

As package warpage increases, the contact pressure at the bonding interface and the load on the interconnects become non-uniform [17,20,58,62]. At some joints, insufficient pressure causes incomplete bonding, while at other locations excessive compressive or shear stress can act. During subsequent thermal cycling, repeated package bending and stress redistribution concentrate tensile and shear stress at particular interconnects and interfaces, and crack growth and delamination tend to progress preferentially in regions where initial defects already exist. Furthermore, because interfacial delamination reduces local stiffness and further concentrates package deformation, warpage and delamination can form a mutually amplifying relationship.

In this way, the failure propagation of HBM must be understood as an interconnected problem involving hotspots, interfacial thermal resistance, current crowding, electromigration, warpage, and delamination. Consequently, it is difficult to adequately assess long-term reliability from a single measurement of thermal conductivity, adhesion strength, or electrical resistance alone. Reliability assessment of next-generation HBM packages requires an experimental approach that combines temperature cycling, power cycling, electromigration testing, warpage measurement, and interfacial fracture analysis, together with coupled multiphysics analysis that simultaneously accounts for thermal, mechanical, and electrical fields [19,25,28,78].

### 2.6. Generational Evolution of HBM Packaging: From Micro-Bump Interconnects to Hybrid Bonding

Since its introduction, HBM has evolved through successive generations—HBM1, HBM2, HBM2E, HBM3, and HBM3E—each of which has increased stack count, per-pin data rate, and I/O density while progressively narrowing the micro-bump pitch used to interconnect the stacked DRAM dies [1,5,23,46,47]. Across these conventional generations, vertical die-to-die connection has relied predominantly on solder-based micro-bumps combined with capillary underfill or NCF, and the dominant packaging issues have evolved together with this pitch scaling. In earlier, lower-count HBM1/HBM2 stacks, the principal concerns were solder-joint thermal-fatigue life, intermetallic-compound growth at the bump interface, and underfill voiding at comparatively relaxed bump pitches [64,65,66,67,68,79]. As stacking increased to 8–12 dies in HBM2E and HBM3/HBM3E and the micro-bump pitch was reduced toward the sub-40 µm range, current crowding, electromigration-driven void formation, and Kirkendall voiding at the solder interface became progressively more severe, while capillary filling of underfill or NCF between the narrower, taller bump arrays became increasingly prone to voids and unfilled regions [24,59,66,67,80]. At the same time, the repeated stacking of micro-bump/underfill layers accumulated interfacial thermal resistance and CTE-mismatch-driven warpage across the die stack, compounding the thermal and mechanical reliability issues discussed in Section 2.2 and Section 2.3 [16,17,18,19,20,44,51].

As the micro-bump pitch approaches its practical scaling floor—set by solder-volume control, bump-to-bump alignment tolerance, and underfill gap-filling capability—hybrid bonding has emerged as the leading interconnect candidate for HBM4 and beyond, eliminating solder and underfill altogether by forming direct Cu–Cu metallic bonds and dielectric–dielectric bonds on a single, planarized interface [1,5,7,23,29,30]. This transition removes the bump-and-fillet geometry that limits micro-bump pitch scaling and enables interconnect pitches well below the practical floor of solder-based joints, together with lower parasitic resistance and capacitance [5,29,30,81]. However, it introduces a qualitatively different set of packaging challenges from those of the micro-bump generations: bonding quality now depends on nanoscale surface flatness, roughness, and cleanliness rather than on solder-reflow behavior, so native-oxide formation, organic contamination, and CMP-induced Cu dishing, erosion, and non-uniform Cu protrusion directly govern void formation and bond strength [21,28,69,70,71,72,73,74,75,76,82]. Achieving a sufficiently low bonding temperature to limit residual stress and CTE-mismatch-driven warpage, while still securing adequate Cu interdiffusion and dielectric bond strength, further requires close control of surface activation, Cu crystallographic orientation, and post-bond annealing [60,61,74,75,83,84,85,86]. Wafer-to-wafer and die-to-wafer bonding also demand tighter overlay alignment and particle control than micro-bump assembly, because a single particle or void can occlude a much larger fraction of the drastically smaller bond pad [25,28,76]. Section 3 and Section 4 examine, respectively, how these package-level and hybrid-bonding-specific interconnect-level issues are being addressed at the material and process level.

## 3. Package-Level Thermal Management Materials

### 3.1. Thermal Interface Materials

A thermal interface material (TIM) is positioned between the HBM or logic die and the heat spreader, where it fills the microscale gaps between the two surfaces and lowers the contact thermal resistance [32,34,39]. Because microscale roughness inevitably exists on the actual chip and heat-spreader surfaces, direct contact results in contact only at certain protrusions, leaving air in the remaining regions. Since air has very low thermal conductivity, these microscale gaps substantially degrade overall heat-transfer performance.

**Figure 5:**
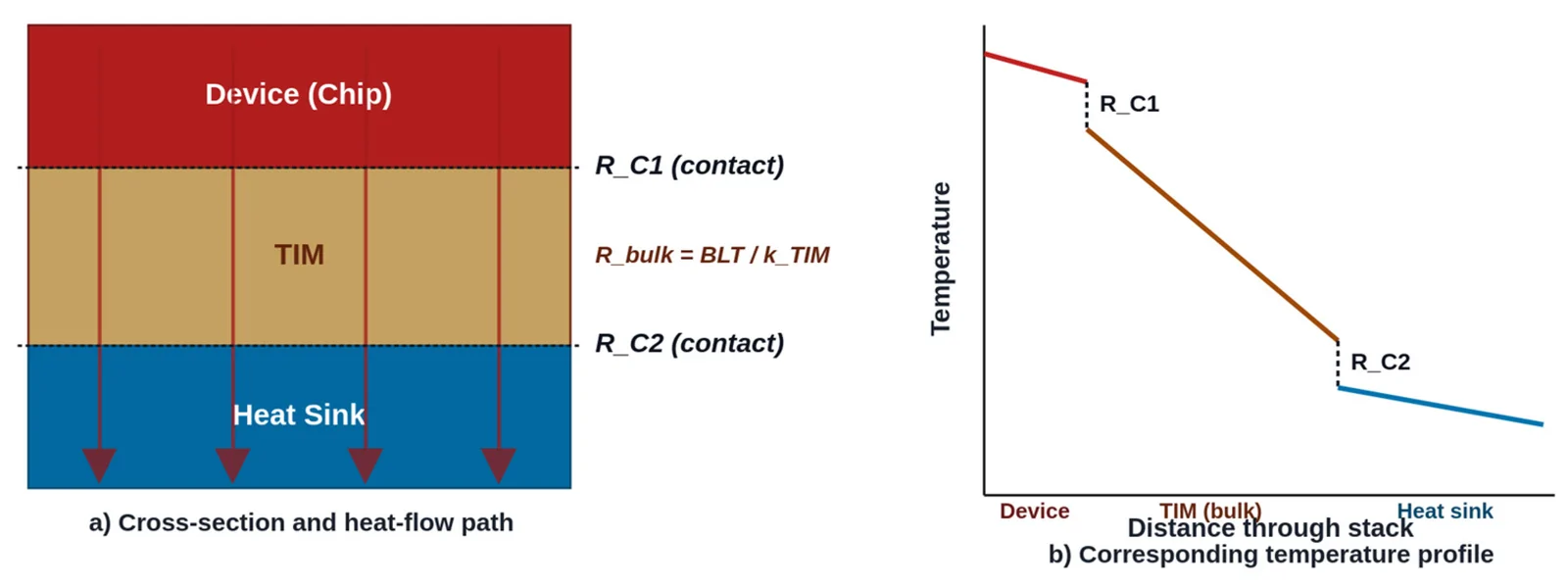
Thermal resistance components of a TIM layer [36]. *Original schematic prepared by the authors.*

As shown in Figure 5, the total thermal resistance through a TIM consists of the bulk thermal resistance that arises within the TIM material itself and the contact thermal resistance that arises at the two interfaces on either side [31,53,59]. The bulk thermal resistance within the TIM is determined by the bond line thickness (BLT) and the thermal conductivity of the TIM, kTIM, and can be expressed as follows.

(1) $$R_{bulk}=\frac{BLT}{k_{TIM}}$$

Here, BLT denotes the thickness of the TIM layer formed between the device and the heat sink. In addition, RC1 denotes the contact thermal resistance at the device–TIM interface, and RC2 denotes the contact thermal resistance at the TIM–heat-sink interface. The effective thermal resistance of the TIM joint can therefore be expressed as follows.

(2) $$R_{TIM,eff}=R_{C1}+\frac{BLT}{k_{TIM}}+R_{C2}$$

As shown in the equation above, the effective thermal resistance of a TIM is determined by the sum of the bulk thermal resistance of the material itself and the contact resistance arising at the chip–TIM and TIM–heat-spreader interfaces [52,87]. Consequently, even a material with high thermal conductivity can exhibit high thermal resistance in an actual package if its surface wetting and conformability are insufficient. Conversely, even a material with relatively low bulk thermal conductivity can effectively lower the overall thermal resistance if a thin bond line thickness and good interfacial contact are secured.

Silicone grease offers excellent flowability and surface conformability, but pump-out and dry-out can occur over long-term use [10,37,88,89]. Phase-change material softens above a certain temperature to improve interfacial contact, but repeated thermal cycling can cause shape-stability and relocation issues. Solder TIM provides high thermal conductivity, but its high elastic modulus and CTE mismatch can transmit large stresses to the chip and heat spreader.

Carbon-based TIMs using CNTs, graphene, and graphite have recently been actively studied [36,57,80,90,91,92]. Owing to their high aspect ratio, CNTs can form a continuous heat-transfer network even at low loading, and vertically aligned CNT structures are advantageous for through-plane heat transfer. However, the junction resistance between CNTs and the polymer–CNT interfacial thermal resistance can limit the actual improvement in thermal conductivity.

Graphene, owing to its high in-plane thermal conductivity, is advantageous for spreading a local hotspot over a wide area, and graphite sheets can likewise be used as large-area heat-spreading materials. However, graphene and graphite exhibit structurally high thermal-conductivity anisotropy, so when platelet fillers align parallel to the interface, through-plane heat-transfer performance can be limited [87,90,92,93]. Therefore, when applying these materials as TIMs, filler orientation, aspect ratio, and interfacial bonding condition must be controlled to secure a vertical heat-transfer pathway. A hybrid network that combines the vertical heat-transfer characteristics of CNTs with the lateral heat-spreading characteristics of graphene or graphite has been proposed as an effective design strategy that complements the directional heat-transfer characteristics of each component and forms a three-dimensional heat-transfer pathway.

A TIM for HBM must simultaneously secure not only high bulk thermal conductivity but also a low and uniform bond line thickness, excellent wetting and conformability, low elastic modulus, resistance to pump-out and dry-out, electrical insulation, and long-term thermal stability [10,32,34,89]. In particular, appropriate viscoelastic behavior and compressive conformability are required to maintain continuous interfacial contact even when the pressure and surface profile between the HBM stack and lid are non-uniform. Accordingly, the design of next-generation HBM TIMs must integrally consider not only the thermal conductivity of the filler itself but also filler-network orientation, polymer–filler interfacial thermal resistance, porosity, bond-line stability, and thermal-cycling reliability.

### 3.2. Underfill and Non-Conductive Film

**Figure 6:**
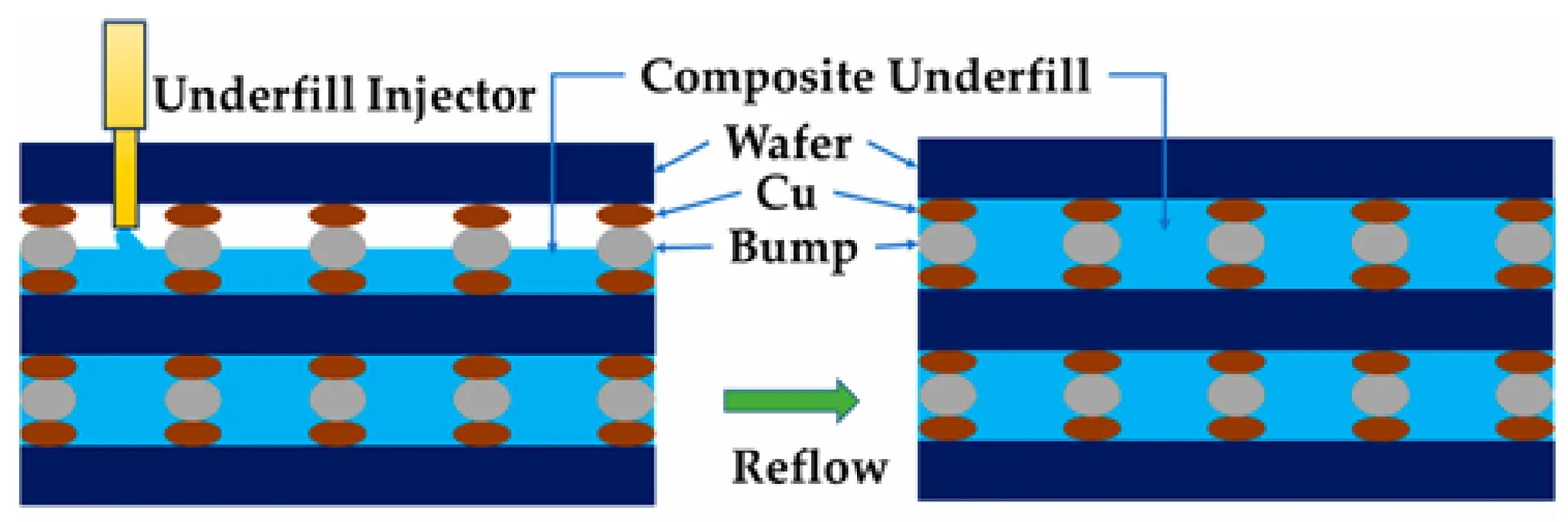
Capillary underfill process and underfill layers in HBM stacking [7]. *Reproduced from Ref.* [7], *an open-access article published in Electronics* (*MDPI*) *under the terms of the Creative Commons Attribution* (*CC BY*) *license.*

Underfill fills the space between the die and the substrate, or between stacked dies, mechanically bonds the die to the substrate, and redistributes the thermo-mechanical strain that would otherwise concentrate at the interconnects across the entire resin layer [37,44,64,79]. It was traditionally used mainly as a reinforcing material to improve the thermal-fatigue life of solder joints, but in highly stacked structures such as HBM, because it is a repeatedly placed interfacial layer, it is also important as a material that constitutes the vertical heat-transfer path. The mechanical reinforcing effect of underfill is determined by the material’s elastic modulus, CTE, adhesion strength, and cure shrinkage; an excessively high elastic modulus can concentrate local stress at die corners or interfaces. This capillary underfill process and the resulting underfill layer structure are illustrated in Figure 6.

Underfill generally consists of epoxy resin, curing agent, catalyst, and inorganic filler. Silica filler is widely used to lower the effective CTE and cure shrinkage of the resin and to improve dimensional stability [64,94,95]. However, as filler content increases, viscosity and elastic modulus increase together, which can restrict capillary flow into fine-pitch interconnects. In particular, as the interconnect pitch and standoff height of HBM decrease, not only the average filler size but also the maximum particle size and particle-size distribution must be precisely controlled.

Using thermally conductive ceramic fillers such as AlN, BN, and Al_2_O_3_ can improve the thermal conductivity of underfill [33,40,96,97]. AlN provides high thermal conductivity together with electrical insulation, and BN can form an efficient heat-transfer network by exploiting its platelet shape. However, because viscosity and elastic modulus also increase with filler content, a trade-off arises between thermal conductivity and processability. Representative thermal and dimensional properties of such AlN–BN/epoxy underfill composites are summarized in Table 1.

In thermally conductive underfill, the type and shape of the filler substantially change the degree to which a heat-transfer network forms even at the same loading level. In particular, combining ceramic fillers of different shapes and sizes allows smaller or platelet-shaped particles to bridge the empty spaces between larger particles, increasing filler–filler contact and forming a more continuous heat-transfer pathway [17,77,78].

Lee Sanchez et al. fabricated an epoxy composite combining spherical AlN particles with platelet BN filler and analyzed the thermal conductivity and thermal-expansion characteristics as a function of filler composition. The thermal conductivity of neat epoxy was approximately 0.22 W·m^−1^·K^−1^, whereas a composite containing 75 wt% AlN/BN hybrid filler reached 10.18 W·m^−1^·K^−1^, an approximately 46-fold improvement in thermal conductivity [64]. In addition, the CTE of this composition decreased to approximately 22.56 ppm·°C^−1^, showing that the introduction of hybrid filler can contribute to improved dimensional stability as well as heat-transfer performance [64].

This performance improvement can be interpreted as arising because AlN forms the primary heat-transfer pathway while the high-aspect-ratio BN platelets bridge the disconnected regions between AlN particles [17,64]. Similarly, an epoxy composite using Al_2_O_3_ together with BN has been reported to show improved thermal conductivity relative to composites using a single filler, owing to enhanced connectivity between particles [77]. Modifying the BN surface with polymer also improves interfacial bonding and dispersion between the filler and the epoxy matrix, allowing electrical insulation to be maintained together with improved thermal conductivity [78].

However, thermal conductivity does not continue to increase proportionally with filler content. Beyond a certain loading level, particle agglomeration and incomplete wetting can increase the interfacial thermal resistance between fillers, and void formation within the polymer matrix can also limit the effective thermal conductivity [17,47,80]. Therefore, achieving high thermal conductivity requires controlling particle-size distribution, particle shape, dispersion state, and the matrix–filler interface together, rather than relying on filler loading alone [17,78].

High filler loading also increases resin viscosity and elastic modulus, which can impede capillary flow between fine-pitch interconnects. In structures such as HBM, where interconnect pitch and die spacing are especially small, filler trapping, unfilled regions, and voids become more likely, and these defects can simultaneously cause local stress concentration and increased interfacial thermal resistance [3,82]. Accordingly, the optimal composition of underfill for HBM should not be the filler content that yields the highest thermal conductivity, but rather the composition that minimizes overall thermal resistance while satisfying the gap-filling capability and processability required at the target gap.

In summary, underfill design using hybrid ceramic fillers is an effective approach for simultaneously improving thermal conductivity and CTE, but practical application in HBM requires jointly considering the trade-offs among filler loading, viscosity, elastic modulus, and void formation [3,47,64]. A strategy of forming a continuous heat-transfer pathway at low filler content using a hybrid filler network of different sizes and shapes combined with surface modification is therefore more appropriate than simply adding a high content of a single filler [17,77,78].

**Figure 7:**
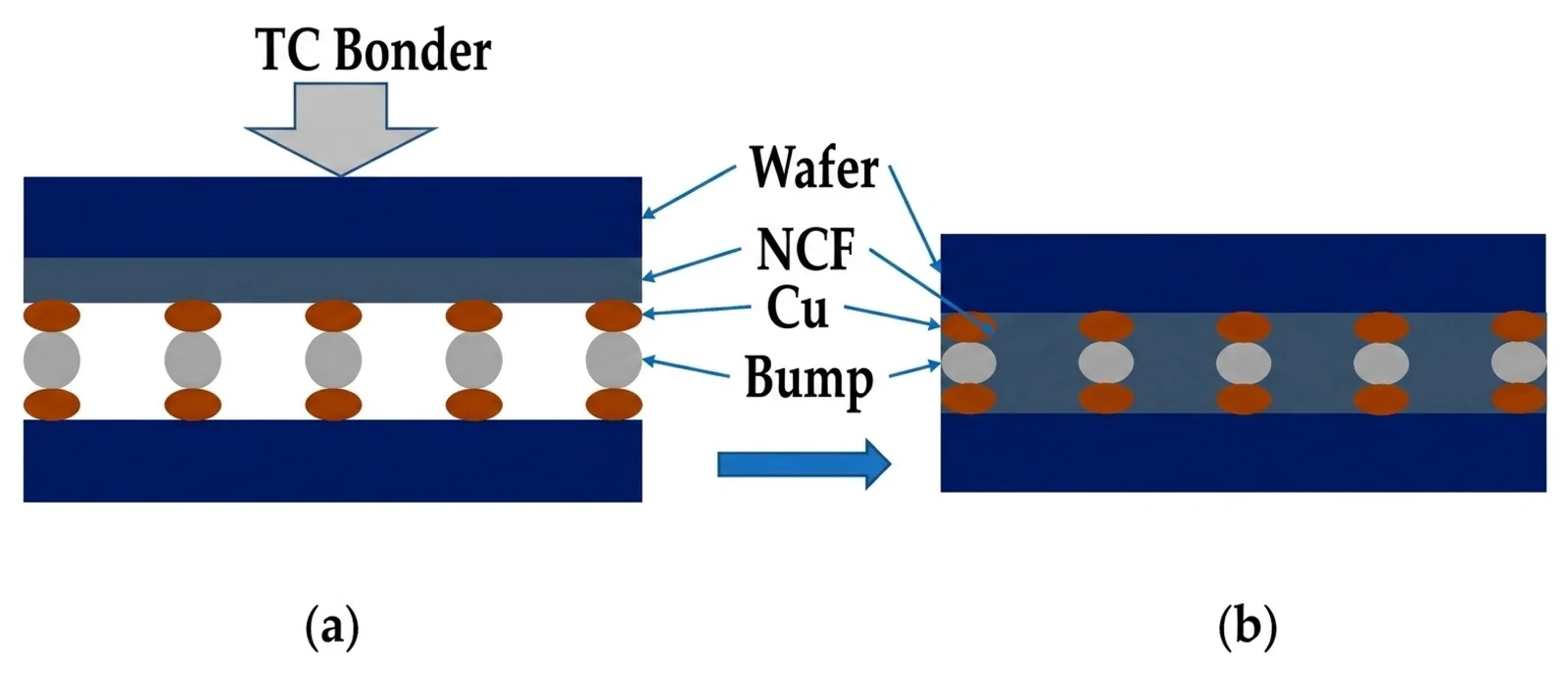
Thermo-compression (TC) bonding process: (**a**) before bonding; (**b**) after bonding [7]. *Reproduced from Ref*. [7], *an open-access article published in Electronics* (*MDPI*) *under the terms of the Creative Commons Attribution* (*CC BY*) *license.*

NCF is pre-placed on the die in film form and softens during the thermo-compression bonding (TCB) process to fill the region around the interconnects [51,72,80]. NCF can shorten the number of process steps and the process time, but if resin flow and filler movement are insufficient during bonding, voids and filler trapping can occur around the bumps. Therefore, viscosity change, curing kinetics, bonding pressure, and heating profile must be optimized together. The corresponding thermo-compression bonding sequence, before and after bonding, is shown in Figure 7.

In highly stacked HBM, because underfill or NCF layers are repeated between each die, the thermal conductivity and thickness of each individual layer accumulate into the overall vertical thermal resistance of the stack [8,44,51]. These materials should therefore be designed not merely as adhesives or reinforcing agents but as functional interfacial layers that simultaneously control heat transfer and stress distribution.

### 3.3. Epoxy Molding Compound and Structural Materials

Epoxy molding compound (EMC) protects the HBM stack and surrounding interconnects from external impact, moisture, and environmental contamination, and secures the overall structural stability of the package [2,63]. However, because EMC occupies a relatively large volume within the package and has a CTE that differs from those of Si, Cu, the interposer, and the organic substrate, it can substantially affect the package warpage and residual stress that develop during fabrication and operation. In particular, in highly stacked HBM, the asymmetric structure and thermal-shrinkage behavior introduced by the EMC can alter the bending deformation and interfacial stress distribution of the entire stack.

A high loading of silica filler is generally added to lower the CTE and cure shrinkage of EMC [20,38,63]. Silica filler is effective in improving the dimensional stability of the resin and suppressing deformation caused by temperature change, but as filler loading increases, viscosity and elastic modulus rise, which can degrade molding flow and the ability to fill fine spaces. Excessive filler content can also increase process pressure and cause filler segregation and local resin starvation. In thin, large-area advanced packages, non-uniform flow of the molding compound, curing reaction, and cooling shrinkage can act together to cause die shift, voids, local thickness variation, and package warpage.

The mechanical properties of EMC must strike a balance between stress relaxation and structural support within the package. If the elastic modulus of the EMC is too high, external loads and thermal deformation can be transmitted directly to the die and interconnects. Conversely, if the modulus is too low, structural support and warpage-suppression capability can become insufficient. Accordingly, EMC design requires integrated optimization among CTE, modulus, glass-transition temperature, cure shrinkage, and moisture absorption [20,62,63].

The interposer and heat spreader likewise affect overall thermal management and mechanical stability [2,12,41]. A silicon interposer is advantageous for fine-line wiring and TSV integration, but it has high brittleness and can develop stress due to CTE mismatch with the organic substrate and molding material. A heat spreader spreads local heat over a wide area, but because it can increase the thermal resistance of the bonding layer and the structural stiffness, its shape and material selection are important.

### 3.4. Multifunctional Packaging Composites

The reliability problems of HBM packages arise from the coupling of thermal bottlenecks, CTE mismatch, warpage, delamination, and interconnect degradation [2,98,99]. Accordingly, next-generation package-level materials are moving away from conventional single-purpose materials that address only one function, toward multifunctional packaging composites that simultaneously secure heat-transfer performance, mechanical reliability, electrical insulation, and long-term stability. In particular, as AI accelerators and HBM become integrated at increasingly high density, heat accumulation and electromagnetic-interference issues can increase together, requiring material designs that integrally consider thermal management alongside structural and electrical function.

A representative approach is to introduce highly thermally conductive fillers into the polymer matrix, TIM, or underfill to form a continuous heat-transfer pathway [57,90,91,92,93]. CNTs and graphene have high intrinsic thermal conductivity and large aspect ratio; vertically aligned CNTs are advantageous for through-plane heat transport, while graphene and graphite are advantageous for lateral heat spreading. A hybrid network combining the vertical heat-transfer characteristics of CNTs with the lateral heat-spreading characteristics of graphene or graphite can therefore simultaneously assist the vertical heat dissipation and hotspot spreading of HBM.

However, as filler loading increases, thermal conductivity and dimensional stability can improve, but viscosity and elastic modulus also increase, which can degrade processability and stress-relaxation performance [10,34,42,95]. A high modulus can be advantageous for suppressing interconnect deformation, but it can also increase the peel and shear stress transmitted to the interface during thermal cycling, raising the risk of delamination.

Three-dimensional thermal networks such as graphene foam, aligned CNT arrays, and CNT/graphene hybrid frameworks have attracted attention as a way to mitigate this trade-off [57,92,93,100]. Introducing a pre-connected heat-transfer skeleton into the polymer matrix can form a continuous thermal pathway without excessive filler loading, while maintaining a certain degree of polymer compliance. This is also advantageous for reducing the filler agglomeration and junction resistance that arise in randomly dispersed structures.

Multifunctional underfill is likewise an important research direction [40,44,64,94,96]. In highly stacked HBM, because underfill is repeatedly placed between each die, it functions simultaneously as a stress-relaxation material and as part of the vertical heat-transfer pathway. Thermally conductive underfill must therefore mitigate local heat accumulation while also effectively redistributing the stress that develops during thermal cycling. However, because increased filler content raises viscosity and flow resistance, which can cause voids and filler trapping in fine-pitch structures, filler size, morphology, and resin rheology must be optimized together.

Electrical insulation is also an important design factor [68,98,99,100]. CNTs, graphene, and graphite can provide high thermal conductivity and electromagnetic-interference shielding performance, but if an electrically conductive network forms, the risk of leakage current and short circuits increases. It is therefore necessary to use carbon fillers with an insulating coating, or ceramic–carbon hybrid fillers combined with BN, AlN, or Al_2_O_3_, to maintain the heat-transfer pathway while suppressing electrical percolation.

The performance of a multifunctional composite is determined not only by the intrinsic properties of the filler but also by its dispersion state, orientation, interfacial bonding, and network connectivity [2,38,42]. Long-term degradation phenomena such as pump-out and dry-out in TIM, and cure shrinkage, moisture absorption, and viscoelastic relaxation in underfill and EMC, must also be considered together.

As a result, next-generation package-level materials for HBM must move beyond simply adding high-thermal-conductivity fillers, toward the integrated design of the filler network, polymer matrix, curing behavior, and interfacial adhesion. The key lies not in maximizing individual properties but in simultaneously controlling the pathways along which heat and stress are transmitted within the HBM structure, which connects directly to the interconnect-level material strategies and interface-oriented material design discussed in the following section.

Table 2 reorganizes the representative material classes, key performance indicators, and principal trade-offs discussed in Section 3.1, Section 3.2, Section 3.3 and Section 3.4 into a single comparative summary.

## 4. Hybrid-Bonding-Centered Interconnect Materials and Interface Engineering for Next-Generation HBM4+ Packages

This chapter focuses specifically on the interconnect-level material and interface technologies required for next-generation HBM4 and beyond, in which hybrid bonding is expected to replace solder-based micro-bumps as the primary die-to-die interconnect. Because hybrid bonding eliminates solder and underfill and instead forms simultaneous Cu–Cu metallic bonding and dielectric–dielectric bonding on a single, planarized interface, it removes the bump-and-fillet geometry that limits micro-bump pitch scaling and allows the interconnect pitch to be reduced well below the practical floor of solder-based joints [1,5,7,23,29,30]. Section 4.1 first summarizes the fine-pitch reliability issues that remain relevant to conventional micro-bump interconnects as a baseline, and Section 4.2, Section 4.3, Section 4.4 and Section 4.5 then examine, in turn, the Cu–Cu and dielectric–dielectric bonding mechanism, low-temperature bonding and residual-stress control, barrier/passivation and dielectric interface design, and interfacial defect control and reliability assessment that together determine the reliability of hybrid-bonded HBM4+ interconnects. Table 3 summarizes representative results from the hybrid-bonding literature reviewed in this chapter.

### 4.1. Fine-Pitch Interconnect Materials

In fine-pitch interconnects, as the joint size decreases, the effect of metal microstructure and interfacial reactions on overall reliability increases. Solder used in micro-bumps forms intermetallic compounds during the bonding process, and as bump size decreases, the fraction of the joint occupied by intermetallic compounds increases [1,65,68]. Excessive growth of intermetallic compounds can cause brittle fracture and increased electrical resistance.

Electromigration is a phenomenon in which the momentum of electrons at high current density is transferred to metal atoms, causing atomic migration. In fine-pitch interconnects, because current is concentrated into a narrow joint, sensitivity to electromigration increases [24,59,67]. Because elevated temperature accelerates atomic diffusion, reliability degradation can progress rapidly in regions where hotspots and current crowding occur simultaneously.

Mitigating this requires controlling Cu microstructure to suppress grain-boundary diffusion and stabilizing interfacial reactions. Cu with large grains and a specific crystallographic orientation can reduce diffusion pathways and improve electromigration resistance [45,61,82]. Barrier and passivation layers can also improve long-term interfacial stability by suppressing Cu diffusion and oxidation.

### 4.2. Cu–Cu and Dielectric–Dielectric Hybrid Bonding

Hybrid bonding simultaneously forms Cu–Cu metallic bonding and dielectric–dielectric bonding on a single plane [1,7,23,29,30,81]. The dielectric surface maintains the initial bonding and alignment condition, and during subsequent thermal treatment, Cu atoms diffuse along the interface, strengthening the metallurgical bond.

**Figure 8:**
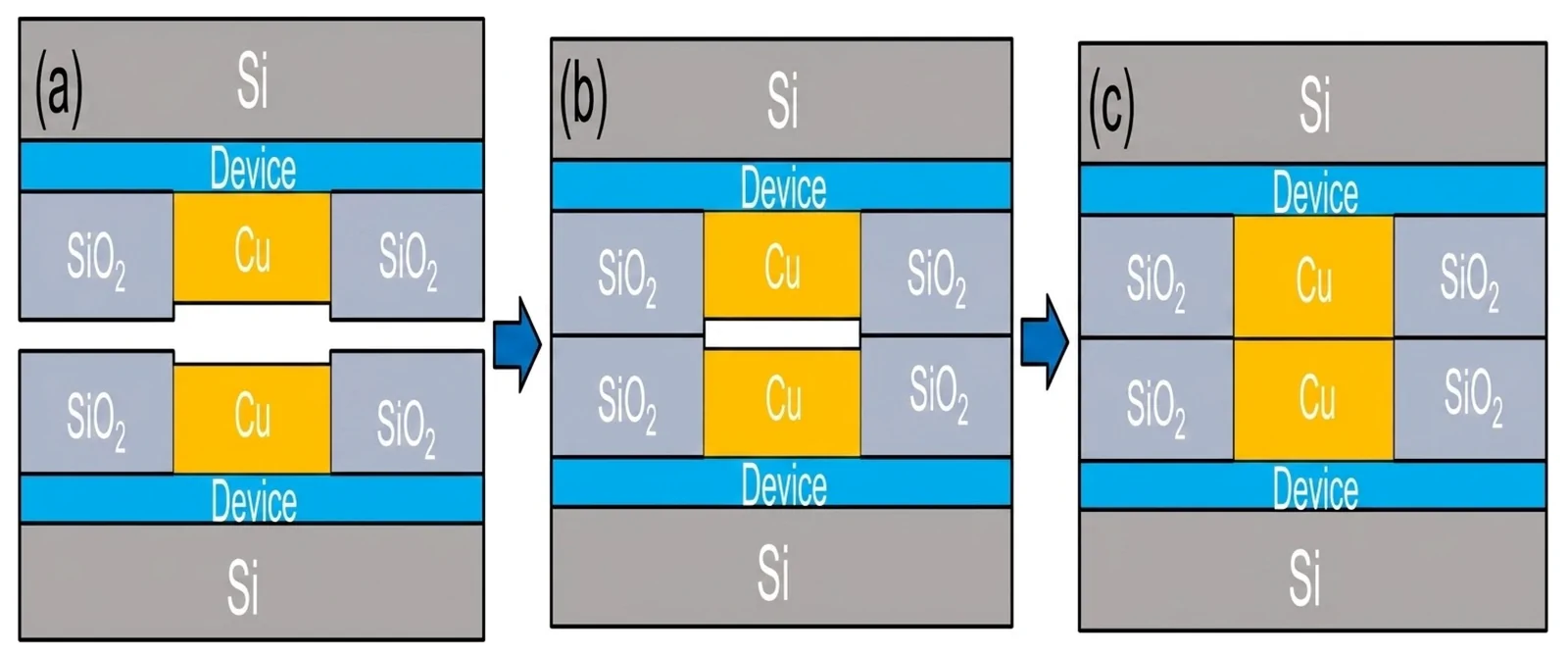
(**a**) two Si/device wafers with SiO_2_-embedded, dished Cu pads prior to bonding; (**b**) initial SiO_2_–SiO_2_ dielectric bonding upon wafer contact, leaving a gap at the recessed Cu–Cu interface;(**c**) Cu–Cu metallic bonding completed after thermal annealing, as thermal expansion of Cu closes the interfacial gap together with the dielectric bond. Schematic illustration of the Cu–Cu and dielectric–dielectric hybrid bonding process [26]. *Reproduced from Ref. [26], an open-access article published in Materials (MDPI) under the terms of the Creative Commons Attribution (CC BY) license.*

To form Cu–Cu direct bonding, the two Cu surfaces must approach one another at the atomic scale. If surface roughness is large or native oxide is present, the actual contact area decreases and unbonded regions can remain [69,70,71,72,73]. Therefore, surface contamination and native oxide must be minimized through CMP, wet cleaning, plasma activation, and surface passivation. This Cu–Cu and dielectric–dielectric hybrid bonding process is illustrated in Figure 8.

In dielectric–dielectric bonding, surface hydroxyl groups and moisture can play an important role in initial bond formation. Plasma activation forms reactive groups on the surface and increases surface energy, enabling initial bonding even at low pressure [26,27,74]. However, excessive plasma treatment can cause surface damage and increased roughness, so process conditions must be precisely controlled.

The crystallographic orientation and grain size of the Cu pad also affect the bonding temperature and quality. Using a crystallographic orientation with high surface diffusivity, or nanocrystalline Cu, can promote atomic migration and interfacial closure even at relatively low temperature [45,61,75,82]. However, nanocrystalline Cu can exhibit rapid grain growth and surface-shape change during thermal treatment, so thermal stability must also be secured.

### 4.3. Low-Temperature Bonding and Residual Stress Control

Lowering the process temperature of hybrid bonding is important for securing the thermo-mechanical reliability of HBM [18,21,22,83]. A high bonding temperature increases the CTE mismatch between Cu and dielectric and can generate large residual stress and warpage during cooling. It can also cause thermal damage to devices and low-k dielectrics that have already been formed.

Surface activation, control of Cu crystallographic orientation, nanocrystalline Cu, passivation layers, and chemical treatment are used to achieve low-temperature bonding [69,72,73,74,84]. Surface activation increases the initial contact force, and reducing the Cu diffusion distance or providing an active diffusion pathway enables bonding to form, even at low temperature.

However, at low process temperature, insufficient Cu atomic diffusion can leave interfacial voids incompletely removed. Therefore, rather than simply lowering the temperature, bonding pressure, annealing time, surface chemistry, and Cu microstructure must be controlled together [60,76,85]. The key is balancing a lower bonding temperature against securing sufficient interfacial bond strength.

If bonding pressure is excessively high, plastic deformation of the Cu pad and dielectric cracking can occur; if the pressure is too low, surface asperities are not sufficiently deformed and the contact area remains limited [77,85,86]. An appropriate pressure window must therefore be established that accounts for both the yield behavior of Cu and the fracture strength of the dielectric.

### 4.4. Barrier, Passivation, and Dielectric Interface Design

Because Cu is sensitive to oxidation and diffusion, barrier and passivation technologies are important for securing interfacial stability before and after the hybrid-bonding process [69,70,71,72,83]. A thin metallic or organic passivation layer can suppress Cu oxidation during storage and alignment, and it must not impede interfacial diffusion or be removed prematurely during bonding.

The barrier layer suppresses Cu diffusion into the surrounding dielectric or silicon [28,77]. However, an increase in barrier thickness can increase both electrical and thermal resistance, so a continuous film must be formed at minimal thickness. In addition, if the interfacial adhesion between the barrier and the Cu and dielectric is insufficient, delamination can occur during thermal cycling.

The dielectric material must simultaneously provide a low dielectric constant, high dielectric breakdown strength, and good bondability [26,27,74]. Increasing porosity to lower the dielectric constant can decrease elastic modulus and interfacial adhesion strength and increase moisture absorption, which can degrade long-term reliability. Dielectric selection should therefore consider mechanical strength, surface chemistry, and thermal stability together, rather than electrical performance alone.

### 4.5. Interfacial Defect Control and Reliability Assessment

Representative defects that arise at the hybrid-bonding interface include voids, particle-induced gaps, oxide residue, misalignment, and local delamination [21,25,28,76]. These defects not only increase electrical contact resistance but also cause disruption of the heat-transfer pathway and stress concentration.

The impact of an interfacial defect depends on its location as well as its size. A void at the center of a Cu pad reduces the effective current-carrying area, while a defect at the pad edge can simultaneously increase current crowding and stress concentration [25,77]. A void at the dielectric interface can act as a moisture-ingress pathway and a crack-initiation site.

Accordingly, the reliability of hybrid bonding should not be assessed from average bond strength alone [25,28,76]. Electrical resistance, thermal resistance, void distribution, fracture mode, and interfacial changes after thermal cycling must be analyzed together. Furthermore, because individual defects are extremely small in submicron-pitch structures, high-resolution non-destructive analysis must be combined with cross-sectional analysis.

Table 3 summarizes representative process and material strategies reported for Cu–Cu and dielectric–dielectric hybrid bonding and their key outcomes, drawn from the studies reviewed in Section 4.2, Section 4.3, Section 4.4 and Section 4.5.

## 5. Integrated Thermal-Reliability Design Strategies

The reliability challenges described in Section 2, Section 3 and Section 4 are not independent: thermal bottlenecks, CTE-mismatch-driven stress, and current-crowding-driven electrical degradation are coupled through shared interfaces, so improving a single material property or a single structural element in isolation cannot resolve the thermal-reliability problem of HBM as a whole. This section therefore examines four complementary design levers—structural thermal-path engineering (Section 5.1), the thermal-conductivity/mechanical-reliability trade-off in packaging materials (Section 5.2), process–material–structure integration (Section 5.3), and interface-oriented material design (Section 5.4)—and, through the key result summarized at the end of each subsection, shows why these levers must ultimately be combined within a single multiphysics thermal-reliability co-design framework (Section 5.5) that simultaneously evaluates the thermal, mechanical, and electrical fields of the package.

### 5.1. Structural Thermal Path Engineering

In highly stacked HBM, improving the thermal conductivity of packaging materials alone is insufficient to fully resolve internal heat accumulation. Thermal path engineering is therefore needed to structurally shorten the path from the heat source to the cooling surface or to form additional heat-transfer channels [8,31,35,54,55].

A thermal TSV is a metal structure placed primarily for heat transfer rather than for electrical connection. Adding thermal TSVs at appropriate locations can rapidly transfer heat from within the stack toward the interposer or heat spreader [48,49,54,55]. However, because increased TSV density affects die area, signal routing, and thermo-mechanical stress, the thermal benefit must be weighed against the design cost.

Placing high-thermal-conductivity dummy dies or thermal bridges around the HBM can spread local heat to the heat spreader [41]. A structure such as a finned lid increases the surface area in contact with the cooling fluid, improving external convective heat transfer. Such structures can be relatively easily combined with existing packaging processes, but the effect of lid stiffness and bonding pressure on package warpage must be evaluated.

Embedded liquid cooling, which forms microchannels within the interposer or heat spreader, has the advantage of removing heat directly at a location close to the heat source [9,35,43]. Compared with conventional approaches that transfer heat to an external heat sink, it can substantially lower the overall thermal resistance, but it introduces new reliability concerns such as channel fabrication, pumping power, leakage, and corrosion.

Structure-based cooling technologies should be optimized together with TIM, underfill, and hybrid bonding rather than applied in isolation [8,9,41,43]. Even if the internal heat-transfer path is improved, overall cooling performance can remain limited if the thermal resistance at the chip–lid interface is high; conversely, even excellent TIM performance may be insufficient to remove a hotspot if a thermal bottleneck exists within the stack.

Key insight: no single structural cooling element—thermal TSV, dummy die/finned lid, or embedded liquid channel—can be optimized independently of the chip-level TIM and bonding interfaces with which it operates in series; the achievable reduction in peak junction temperature is set by whichever stage of the heat-transfer path is least optimized, not by the best-performing stage alone.

### 5.2. Thermal Conductivity–Mechanical Reliability Trade-Off

Increasing filler content to raise the thermal conductivity of a packaging material forms a continuous heat-transfer network within the polymer matrix [10,33,34,42]. However, viscosity, elastic modulus, and brittleness can increase at the same time. High viscosity impedes filling between narrow interconnects, and a high modulus transmits temperature-induced deformation directly to the interface.

Accordingly, in filler design, it is important to establish the minimum filler content required to achieve a target thermal resistance, rather than maximizing thermal conductivity. Hybrid fillers, filler alignment, and surface modification can be used to form an efficient heat-transfer pathway without excessive filler loading [40,57,96,100].

If the interfacial bonding between the thermally conductive filler and the polymer is too weak, phonon scattering and interfacial thermal resistance increase [52,68,97]. Conversely, if the interfacial bonding is excessively strong and rigid, stress-relaxation capability can decrease. An appropriate level of interfacial bonding that considers both heat transfer and mechanical damping is therefore required.

Optimization of HBM materials should not be carried out by independently maximizing or minimizing individual values such as thermal conductivity, CTE, and modulus. Because these properties are interrelated, it is more appropriate to establish a property window suited to the package geometry and process conditions [2,37,38].

Key insight: because thermal conductivity, viscosity, elastic modulus, and interfacial adhesion move together as filler content changes, the useful design variable is not the maximum achievable thermal conductivity but the minimum filler loading that meets a target thermal-resistance specification without violating the gap-filling and stress-relaxation limits of the package.

### 5.3. Process–Material–Structure Integration

The manufacturing reliability of HBM is determined by the interaction between material properties and process conditions. Even with the same NCF, resin flow, filler distribution, and degree of cure can vary depending on bonding temperature and pressure [51,63,64,84]. Similarly, even for the same hybrid-bonding surface, the oxide and contamination condition changes depending on the waiting time and environment after cleaning.

Material development must therefore reflect the actual process heating rate, pressure profile, bonding time, and cooling condition from an early stage [18,20,22]. Simple coupon-level property evaluation alone cannot sufficiently predict the resin flow, warpage, and interfacial stress that occur in an actual multilayer stack.

Linking process simulation with structural analysis makes it possible to predict the temperature distribution, cure shrinkage, warpage, and residual stress that develop during manufacturing [10,17,48,58]. Reflecting these analytical results in material formulation and bonding conditions can reduce the reliance on trial-and-error process development.

In addition, because HBM has a structure in which the die, package, and system levels are strongly coupled, the optimal condition for an individual layer may not coincide with the optimal condition for the overall package. For example, a stiffer underfill can reduce local strain at the micro-bump but increase stress at the package edge [19,78,79]. A multi-scale analysis that simultaneously considers various length scales is therefore required.

Key insight: because coupon-level material data cannot reproduce the resin flow, cure kinetics, and warpage that develop in an actual multilayer stack, and because a locally optimal material condition—for example, a stiffer underfill at the micro-bump—can be globally unfavorable by increasing stress at the package edge, reliable process design requires linking process simulation directly to structural analysis rather than relying on independent, single-scale optimization.

### 5.4. Interface-Oriented Material Design

The hotspots, delamination, Cu protrusion, void formation, and bonding failures that occur in HBM may appear to be distinct phenomena, but most are closely related to the characteristics of the interfaces between materials [7,28,39,52]. Heat encounters resistance as it passes through an interface, stress concentrates at interfaces where material properties change discontinuously, and current is likewise affected by contact area and interfacial defects.

Accordingly, next-generation HBM materials should be designed around the actual behavior at the interface rather than around bulk properties alone. For TIM, the wetting and contact resistance with respect to the chip and heat spreader are more important than the thermal conductivity of the filler itself; for underfill, adhesion to Cu, passivation, and dielectric is important in addition to bulk modulus [26,32,37,44]. In hybrid bonding as well, oxide, roughness, grain orientation, and voids determine actual performance more than the intrinsic conductivity of Cu.

Interface-oriented material design is an approach that simultaneously controls interfacial thermal resistance, interfacial adhesion, interfacial stress, and interfacial chemistry [71,72,74,83]. This can be achieved using techniques such as filler surface functionalization, gradient interlayers, compliant interfaces, oxide control, and selective surface activation.

A gradient structure can mitigate the abrupt change in thermal and mechanical properties between two dissimilar materials [88,89]. For example, forming an interfacial layer in which filler content or modulus changes gradually through the thickness can reduce stress concentration while maintaining the heat-transfer pathway. Applying a thin compliant layer can also absorb deformation at the bonding interface and suppress crack initiation.

Key insight: across TIM, underfill, and hybrid-bonding interfaces alike, the properties that dominate package-level performance are interfacial—wetting, contact resistance, adhesion, and interfacial chemistry—rather than the bulk properties of the constituent materials, so interface-oriented design strategies such as gradient interlayers, compliant layers, and surface functionalization yield larger reliability gains than further optimization of bulk filler content alone.

### 5.5. Thermal-Reliability Co-Design

In conventional packaging design, thermal performance, mechanical reliability, and electrical performance have often been evaluated sequentially. In HBM, however, because a single design variable can simultaneously affect multiple performance metrics, thermal, mechanical, electrical, and process characteristics must be integrally considered from the early design stage [6,9,12,45].

In thermal-reliability co-design, material thermal conductivity, CTE, modulus, interfacial adhesion, electrical resistivity, and processing window are simultaneously used as design variables [2,8,15]. Die thickness, stack height, TSV density, interconnect pitch, TIM thickness, and cooling structure must also be optimized together.

For example, increasing thermal-TSV density can improve heat-transfer performance but can also increase silicon area consumption and stress concentration. Increasing the filler content of underfill decreases the stack’s thermal resistance but can increase viscosity and interfacial stress. Lowering the hybrid-bonding temperature decreases residual stress but can result in insufficient interfacial diffusion and bond strength. Co-design is the process of accounting for these trade-off relationships to find the optimal operating window for the overall package, rather than the highest value of any single performance metric [10,35,43,78].

In the future, predictive models that combine multiphysics simulation with experimental reliability data are expected to become increasingly important [3,15,17,22,48]. Jointly computing the temporal evolution of temperature, stress, current density, and interfacial defects enables more accurate prediction of failure initiation and propagation. Machine-learning-based models can also be used to rapidly explore promising design regions across the vast space of material composition and process variables.

Ultimately, the reliability of next-generation HBM must be secured not by independently optimizing package-level materials and interconnect-level interfaces, but through multi-scale interface engineering that connects the two domains [1,5,56]. This represents a design paradigm that treats materials, interfaces, processes, and structure as a single system and simultaneously controls the pathways along which heat, stress, and current are transferred.

## 6. Conclusions

HBM has evolved across successive generations—from HBM1 through HBM3E on solder-based micro-bump interconnects, and now toward HBM4 and beyond on hybrid bonding—with each generation trading a narrower interconnect pitch and a higher stack count for a longer, more resistive heat-transfer path and a more tightly coupled set of thermal and mechanical reliability risks (Section 2.6). Across this transition, the heat-transfer performance of HBM depends less on the thermal conductivity of any individual material than on the interfacial thermal resistance that accumulates at the underfill, NCF, TIM, and bonding interfaces repeated at every die level.

At the package level (Section 3, summarized in Table 2), TIM, underfill/NCF, EMC, and multifunctional composites each show a consistent trade-off in which raising filler content to increase thermal conductivity simultaneously raises viscosity, elastic modulus, and brittleness. The AlN–BN hybrid-filler underfill system in Table 1, which improved thermal conductivity by roughly 46-fold while lowering CTE, illustrates that hybrid, multi-shape filler networks can shift this trade-off favorably, but only within a processability window that must be verified case by case at the target pitch and gap.

At the interconnect level (Section 4), the transition from micro-bump interconnects to hybrid bonding for HBM4 and beyond removes the pitch-limiting bump geometry but introduces a fundamentally interfacial reliability problem: bonding quality is now governed by nanoscale surface flatness, cleanliness, Cu crystallographic condition, and dielectric surface chemistry rather than by solder-reflow metallurgy (Table 3). Achieving low-temperature, low-defect Cu–Cu and dielectric–dielectric bonding therefore requires the coordinated control of CMP-induced Cu protrusion and dishing, surface activation and passivation, and post-bond annealing, rather than the optimization of any single process step in isolation.

These material- and interconnect-level findings converge on the conclusion reached in Section 5: because the thermal, mechanical, and electrical degradation mechanisms of HBM are mutually coupled through shared interfaces, structural thermal-path engineering, filler and material design, process conditions, and interface engineering cannot be optimized independently without shifting the reliability burden elsewhere in the package. Realizing the full benefit of any single improvement—an added thermal TSV, a higher-conductivity underfill, a lower hybrid-bonding temperature—therefore requires evaluating it within a multiphysics thermal-reliability co-design framework that simultaneously tracks temperature, stress, and current density across the package.

Accordingly, next-generation HBM packaging requires multi-scale interface engineering and thermal-reliability co-design that jointly control the heat-, stress-, and current-transfer pathways across both the package and interconnect domains, rather than independently optimizing package-level materials and interconnect-level interfaces. This integrated design strategy, informed by the generational packaging trends, material trade-offs, hybrid-bonding reliability mechanisms, and coupled-design insights summarized above, is expected to serve as a key foundation for securing the thermal management performance and long-term reliability of ultra-highly stacked memory beyond HBM4.

## Figures and Tables

**Table 1:** Representative thermal and dimensional properties of AlN–BN/epoxy underfill composites [64].

Material	Hybrid Filler Loading	Thermal Conductivity	Relative Improvement	CTE
Neat epoxy	0 wt%	0.22 W·m^−1^·K^−1^	1×	–
AlN–BN/epoxy	75 wt%	10.18 W·m^−1^·K^−1^	~46×	22.56 ppm·°C^−1^

**Table 2:** Summary of key package-level thermal management materials discussed in Section 3.

Section/Category	Representative Materials	Key Property or Indicator	Main Advantage	Main Trade-Off/Challenge
3.1 Thermal interface materials (TIM)	Silicone grease; phase-change material (PCM); solder TIM; CNT-, graphene-, and graphite-based TIM	Effective joint resistance R_TIM,eff = R_C1 + BLT/k_TIM + R_C2 (Equations (1) and (2)); bond-line thickness (BLT) and bulk k_TIM	Grease: high flow/conformability; PCM: improved contact above transition temperature; solder: high bulk k; carbon-based: high k at low loading, tunable directionality	Grease: pump-out/dry-out; PCM: shape stability under cycling; solder: high modulus/CTE mismatch stress on chip; carbon-based: junction and polymer–filler interfacial resistance, in-plane/through-plane anisotropy
3.2 Underfill and non-conductive film (NCF)	Silica-filled epoxy underfill; AlN–BN hybrid-filler epoxy underfill (Table 1); NCF	Thermal conductivity rising from 0.22 W·m^−1^·K^−1^ (neat epoxy) to 10.18 W·m^−1^·K^−1^ at 75 wt% AlN–BN loading (~46×), with CTE reduced to ≈22.56 ppm·°C^−1^ [64]	Mechanical reinforcement of interconnects plus a repeated vertical heat-transfer path; hybrid, multi-shape fillers bridge inter-particle gaps for a more continuous network	Viscosity and elastic modulus rise with filler loading, restricting capillary flow and gap-filling at fine pitch; particle agglomeration and voids above the optimal loading
3.3 Epoxy molding compound (EMC) and structural materials	Silica-filled EMC; silicon interposer; metal heat spreader	CTE, cure shrinkage, glass-transition temperature, and elastic modulus as coupled design targets	High silica loading improves dimensional stability and suppresses warpage; heat spreader enables wide-area heat spreading	High filler loading impairs molding flow and fine-feature filling; elastic-modulus trade-off between stress transfer to the die and structural/warpage support
3.4 Multifunctional packaging composites	Vertically aligned CNT arrays; graphene/graphite foams; CNT–graphene hybrid networks; ceramic–carbon hybrid (BN/AlN/Al_2_O_3_ + carbon) fillers	Continuous heat-transfer pathway formed at reduced filler loading; electrical percolation threshold	Decouples thermal conductivity from total filler loading while preserving polymer compliance; can combine thermal management with EMI shielding and electrical insulation	Requires precise control of filler dispersion, orientation, and interfacial bonding; risk of electrically conductive percolation if not insulated or hybridized

**Table 3:** Representative process and material strategies for Cu–Cu/dielectric hybrid-bonding reliability in next-generation HBM interconnects.

Reference(s)	Approach/Technique	Reported Outcome or Key Finding
Ong et al. [26]	Low-temperature Cu/SiO_2_ hybrid bonding using (111)-oriented Cu surfaces	Exploiting Cu crystallographic orientation promoted Cu interdiffusion and achieved low contact resistance at reduced bonding temperature
Kang et al. [27]	Co-hydroxylated Cu/SiO_2_ hybrid-bonding strategy	Enhanced dielectric-surface hydroxylation strengthened initial low-temperature pre-bonding for memory-centric chip architectures
Wang et al. [21]	Cu-protrusion optimization for wafer-to-wafer hybrid bonding	Identified control of Cu protrusion/coplanarity as critical to bonding-interface quality in HBM packages
Zhang et al. [61]; Chiu et al. [75]	(111)-oriented nanotwinned-Cu bonding	Enhanced surface diffusivity of nanotwinned Cu enabled Cu–Cu direct bonding in ambient atmosphere and at reduced temperature
Park et al. [69,70,71,83,84]	Plasma-based surface activation and Cu passivation (N_2_ plasma, copper-nitride nanolayer)	Suppressed native-oxide regrowth and enabled solderless Cu–Cu bonding at temperatures as low as ~260 °C
Hung et al. [72]; Hong et al. [73]	PVD-deposited passivation layers (e.g., Cu_3_N)	Provided low-cost oxidation protection compatible with subsequent low-temperature bonding
Lhostis et al. [25]; Moreau et al. [28]	Reliability assessment of submicrometric hybrid-bonding pads	Identified void distribution and pad-edge defects as the dominant factors limiting bonding-level reliability
Wenzel et al. [76]	Post-bond heat-treatment study for die-to-wafer hybrid bonds	Showed that annealing conditions strongly affect final interfacial bond-interconnect quality
Lu et al. [85]; Wang et al. [86]	Bonding-pressure control and in-situ oxide reduction (e.g., glycerol-assisted)	Controlled compressive stress and chemical oxide reduction each expand the achievable low-temperature bonding process window

## Data Availability

No new data were created or analyzed in this study. Data sharing is not applicable to this article.

## References

[1] Lau J.H. (2021). State-of-the-art and outlooks of chiplets heterogeneous integration and hybrid bonding. J. Microelectron. Electron. Packag..

[2] Pulugurtha M.R., Sharma H., Pucha R., Kathaperumal M., Tummala R. (2022). Packaging materials in high-performance computing applications. J. Indian Inst. Sci..

[3] Chae J.-H. (2024). High-bandwidth and energy-efficient memory interfaces for the data-centric era: Recent advances, design challenges, and future prospects. IEEE Open J. Solid-State Circuits Soc..

[4] Moon K.-I., Son H.-Y., Lee K. Advanced packaging technologies in memory applications for future generative AI era. Proceedings of the 2023 International Electron Devices Meeting (IEDM).

[5] Lee C.Y., Won C.H., Jung S., Jung E.S., Choi T.M., Lee H.R., Yoo J., Yoon S., Pyo S.G. (2025). 3D integrated process and hybrid bonding of high bandwidth memory (HBM). Electron. Mater. Lett..

[6] Kim T., Lee J., Kim J., Lee E.-C., Hwang H., Kim Y., Oh D.K.S. Thermal modeling and analysis of high bandwidth memory in 2.5 D Si-interposer systems. Proceedings of the 2022 21st IEEE Intersociety Conference on Thermal and Thermomechanical Phenomena in Electronic Systems (iTherm).

[7] Lee S.-H., Kim S.-J., Lee J.-S., Rhi S.-H. (2025). Thermal issues related to hybrid bonding of 3D-stacked high bandwidth memory: A comprehensive review. Electronics.

[8] Kim T., Lee J., Kim Y., Park H., Hwang H., Kim J., Jung H., Kim D.W. Thermal improvement of HBM with joint thermal resistance reduction for scaling 12 stacks and beyond. Proceedings of the 2023 IEEE 73rd Electronic Components and Technology Conference (ECTC).

[9] Son K., Kim S., Park H., Shin T., Kim K., Kim M., Sim B., Kim S., Park G., Park S. (2022). Thermal and signal integrity co-design and verification of embedded cooling structure with thermal transmission line for high bandwidth memory module. IEEE Trans. Compon. Packag. Manuf. Technol..

[10] Chatterjee K., Li Y., Chang H., Damadam M., Asrar P., Kim J., Jeong G., Kim W. Thermal and mechanical simulations of 3D packages with custom high bandwidth memory (HBM). Proceedings of the 2024 IEEE 74th Electronic Components and Technology Conference (ECTC).

[11] Son K., Park J., Kim S., Sim B., Kim K., Choi S., Kim H., Kim J. Thermal analysis of high bandwidth memory (HBM)-GPU module considering power consumption. Proceedings of the 2023 IEEE Electrical Design of Advanced Packaging and Systems (EDAPS).

[12] Hu J., Li T., Zhang H., Fan Y. Signal and power integrity co-simulation of HBM interposer in high density 2.5D package. Proceedings of the 2022 23rd International Conference on Electronic Packaging Technology (ICEPT).

[13] Song J.-H., Yoon S. (2025). Comparative study of thermal dissipation in increasing DRAM layers of HBM using 3D FEA simulations. J. Semicond. Technol. Sci..

[14] Chalise D., Cahill D.G. (2023). Anisotropic thermal conductivity of high bandwidth memory. arXiv.

[15] Huang L.-H., Cheng Y.-Y., Wu T.-L. (2024). Analysis and optimization of HBM3 PPA for TSV model with micro-bump and hybrid bonding. IEEE Trans. Compon. Packag. Manuf. Technol..

[16] Jiang T., Im J., Huang R., Ho P.S. (2015). Through-silicon via stress characteristics and reliability impact on 3D integrated circuits. MRS Bull..

[17] Kim H., Hwang J.Y., Kim S.E., Joo Y.-C., Jang H. (2023). Thermomechanical challenges of 2.5-D packaging: A review of warpage and interconnect reliability. IEEE Trans. Compon. Packag. Manuf. Technol..

[18] Jeon J., Song J., Oh J.Y., Lee S.H., Kim Y., Kim Y.H., Choi S.-J., Yoon S.W. (2025). Residual stress and deformation analysis considering adhesive material properties to enhance manufacturing of HBM. J. Mater. Res. Technol..

[19] Lee C.-C., Lin Y.-M., Liu H.-C., Syu J.-Y., Huang Y.-C., Chang T.-C. (2021). Reliability evaluation of ultra thin 3D-IC package under the coupling load effects of the manufacturing process and temperature cycling test. Microelectron. Eng..

[20] Um H.-J., Ju Y.-M., Lee D.-W., Ahn J., Kim H.-S. (2024). Reduced warpage in semiconductor packages: Optimizing post-cure temperature profile considering cure shrinkage and viscoelasticity of epoxy molding compound. Mater. Des..

[21] Wang S., Zhang H., Tian Z., Liu T., Sun Y., Zhang Y., Dong F., Liu S. (2022). Optimization of Cu protrusion of wafer-to-wafer hybrid bonding for HBM packages application. Mater. Sci. Semicond. Process..

[22] Liu Y., Yao C., Sun F., Fang H. (2021). Numerical simulation of reliability of 2.5 D/3D package interconnect structure under temperature cyclic load. Microelectron. Reliab..

[23] Ohba T., Sakui K., Sugatani S., Ryoson H., Chujo N. (2022). Review of bumpless build cube (BBCube) using wafer-on-wafer (WOW) and chip-on-wafer (COW) for tera-scale three-dimensional integration (3DI). Electronics.

[24] Zhou A., Zhang Y., Ding F., Lian Z., Jin R., Yang Y., Wang Q., Cao L. (2024). Research progress of hybrid bonding technology for three-dimensional integration. Microelectron. Reliab..

[25] Lhostis S., Ayoub B., Frémont H., Moreau S., Mattei J.-G., Lamontagne P., Tournier A. (2023). Reliability of the hybrid bonding level using submicrometric bonding pads. Microelectron. Reliab..

[26] Ong J.-J., Chiu W.-L., Lee O.-H., Chiang C.-W., Chang H.-H., Wang C.-H., Shie K.-C., Yang S.-C., Tran D.-P., Tu K.-N. (2022). Low-temperature Cu/SiO_2_ hybrid bonding with low contact resistance using (111)-oriented Cu surfaces. Materials.

[27] Kang Q., Wang C., Zhou S., Li G., Lu T., Tian Y., He P. (2021). Low-temperature Co-hydroxylated Cu/SiO_2_ hybrid bonding strategy for a memory-centric chip architecture. ACS Appl. Mater. Interfaces.

[28] Moreau S., Jourdon J., Lhostis S., Bouchu D., Ayoub B., Arnaud L., Frémont H. (2022). Review—Hybrid bonding-based interconnects: A status on the last robustness and reliability achievements. ECS J. Solid State Sci. Technol..

[29] Bao W., Zhang J., Wong H., Liu J., Li W. (2025). Emerging Copper-to-Copper Bonding Techniques: Enabling High-Density Interconnects for Heterogeneous Integration. Nanomaterials.

[30] Jiang B., Wu Y., Zhong Z., Fan K., Lin Q., Liang J., Liu H., Liu X. (2025). Cu–Cu hybrid bonding technology: From physical mechanisms to system integration for 3D ICs. Moore More.

[31] Sanipini V.K., Rakesh B., Chamanthula A.J., Santoshi N., Gudivada A.A., Panigrahy A.K. (2021). Thermal management in TSV based 3D IC Integration: A survey. Mater. Today Proc..

[32] Wei B., Luo W., Du J., Ding Y., Guo Y., Zhu G., Zhu Y., Li B. (2024). Thermal interface materials: From fundamental research to applications. SusMat.

[33] Meng Y., Yang D., Jiang X., Bando Y., Wang X. (2024). Thermal conductivity enhancement of polymeric composites using hexagonal boron nitride: Design strategies and challenges. Nanomaterials.

[34] Ma H., Gao B., Wang M., Yuan Z., Shen J., Zhao J., Feng Y. (2021). Strategies for enhancing thermal conductivity of polymer-based thermal interface materials: A review. J. Mater. Sci..

[35] Rangarajan S., Schiffres S.N., Sammakia B. (2023). A review of recent developments in “on-chip” embedded cooling technologies for heterogeneous integrated applications. Engineering.

[36] Guo X., Cheng S., Cai W., Zhang Y., Zhang X.-A. (2021). A review of carbon-based thermal interface materials: Mechanism, thermal measurements and thermal properties. Mater. Des..

[37] Alim M.A., Abdullah M.Z., Aziz M.S.A., Kamarudin R., Gunnasegaran P. (2021). Recent advances on thermally conductive adhesive in electronic packaging: A review. Polymers.

[38] Wan Y.-J., Li G., Yao Y.-M., Zeng X.-L., Zhu P.-L., Sun R. (2020). Recent advances in polymer-based electronic packaging materials. Compos. Commun..

[39] Chung D. (2022). Performance of thermal interface materials. Small.

[40] Lee Sanchez W.A., Li J.-W., Chiu H.-T., Cheng C.-C., Chiou K.-C., Lee T.-M., Chiu C.-W. (2022). Highly thermally conductive epoxy composites with AlN/BN hybrid filler as underfill encapsulation material for electronic packaging. Polymers.

[41] Lin H.-C., Tseng C.-W., Chen Y.-Y., Tong H.-M., Liu C.W. High-Thermal-conductivity Dummy Die and Finned Lid for Enhanced Liquid Cooling of 2.5D ICs. Proceedings of the 2024 IEEE 26th Electronics Packaging Technology Conference (EPTC).

[42] Wang J., Hu L., Li W., Ouyang Y., Bai L. (2022). Development and perspectives of thermal conductive polymer composites. Nanomaterials.

[43] van Erp R., Soleimanzadeh R., Nela L., Kampitsis G., Matioli E. (2020). Co-designing electronics with microfluidics for more sustainable cooling. Nature.

[44] Wen Y., Chen C., Ye Y., Xue Z., Liu H., Zhou X., Zhang Y., Li D., Xie X., Mai Y. (2022). Advances on thermally conductive epoxy-based composites as electronic packaging underfill materials—A review. Adv. Mater..

[45] Shen Y., Schreuders L., Pathania A., Pimentel A.D. (2023). Thermal management for 3d-stacked systems via unified core-memory power regulation. ACM Trans. Embed. Comput. Syst..

[46] Wang J., Duan F., Lv Z., Chen S., Yang X., Chen H., Liu J. (2023). A short review of through-silicon via (TSV) interconnects: Metrology and analysis. Appl. Sci..

[47] Lau J.H. (2011). Overview and outlook of through-silicon via (TSV) and 3D integrations. Microelectron. Int..

[48] Tavakkoli F., Ebrahimi S., Wang S., Vafai K. (2016). Analysis of critical thermal issues in 3D integrated circuits. Int. J. Heat Mass Transf..

[49] Cheng H.-C., Huang T.-C., Hwang P.-W., Chen W.-H. (2016). Heat dissipation assessment of through silicon via (TSV)-based 3D IC packaging for CMOS image sensing. Microelectron. Reliab..

[50] Lu K.-H., Ryu S.-K., Im J., Huang R., Ho P.S. Thermomechanical reliability of through-silicon vias in 3D interconnects. Proceedings of the 2011 International Reliability Physics Symposium.

[51] Lee Y., Yoon S.W. Thermal Conductivity Aware Design Approach of Non-Conductive Film Layers for Enhanced Vertical Heat Dissipation in 3D HBM Packages. Proceedings of the 2025 IEEE CPMT Symposium Japan (ICSJ).

[52] Zhan K., Chen Y., Xiong Z., Zhang Y., Ding S., Zhen F., Liu Z., Wei Q., Liu M., Sun B. (2024). Low thermal contact resistance boron nitride nanosheets composites enabled by interfacial arc-like phonon bridge. Nat. Commun..

[53] Chung D. (2023). A critical review of carbon-based thermal interface materials. Mater. Chem. Phys..

[54] Cong J., Zhang Y. Thermal-aware physical design flow for 3-D ICs. Proceedings of the 23rd International VLSI Multilevel Interconnection Conference.

[55] Singh S.G., Tan C.S. Thermal mitigation using thermal through silicon via (TTSV) in 3-D ICs. Proceedings of the 2009 4th International Microsystems, Packaging, Assembly and Circuits Technology Conference.

[56] Rahman A., Reif R. Thermal analysis of three-dimensional (3-D) integrated circuits (ICs). Proceedings of the IEEE 2001 International Interconnect Technology Conference (Cat. No. 01EX461).

[57] Lian G., Tuan C.-C., Li L., Jiao S., Wang Q., Moon K.-S., Cui D., Wong C.-P. (2016). Vertically aligned and interconnected graphene networks for high thermal conductivity of epoxy composites with ultralow loading. Chem. Mater..

[58] Jung M., Pan D.Z., Lim S.K. Chip/package co-analysis of thermo-mechanical stress and reliability in TSV-based 3D ICs. Proceedings of the 49th Annual Design Automation Conference.

[59] Tu K.-N. (2011). Reliability challenges in 3D IC packaging technology. Microelectron. Reliab..

[60] Shie K.-C., Gusak A., Tu K., Chen C. (2021). A kinetic model of copper-to-copper direct bonding under thermal compression. J. Mater. Res. Technol..

[61] Zhang M., Gao L.-Y., Li J.-J., Sun R., Liu Z.-Q. (2023). Characterization of Cu-Cu direct bonding in ambient atmosphere enabled using (111)-oriented nanotwinned-copper. Mater. Chem. Phys..

[62] Ko C.-T., Chen K.-N. (2013). Reliability of key technologies in 3D integration. Microelectron. Reliab..

[63] Othman A.H., Aziz M.S.A., Ishak M.H.H., Nashrudin M.N., Azman M.A. (2026). Epoxy molding compound encapsulation process in IC packaging: A review at wafer and component levels. Int. J. Adv. Manuf. Technol..

[64] Plachý Z., Pražanová A., Dušek K., Géczy A. (2025). Underfill: A review of reliability improvement methods in electronics production. Polymers.

[65] Jang Y.J., Sharma A., Jung J.P. (2023). Advanced 3D through-Si-via and solder bumping technology: A review. Materials.

[66] Cho D.H., Seo S.M., Kim J.B., Rajendran S.H., Jung J.P. (2021). A review on the fabrication and reliability of three-dimensional integration technologies for microelectronic packaging: Through-Si-via and solder bumping process. Metals.

[67] Banijamali B., Ramalingam S., Liu H., Kim M. Outstanding and innovative reliability study of 3D TSV interposer and fine pitch solder micro-bumps. Proceedings of the 2012 IEEE 62nd Electronic Components and Technology Conference.

[68] Morishita T., Okamoto H. (2016). Facile exfoliation and noncovalent superacid functionalization of boron nitride nanosheets and their use for highly thermally conductive and electrically insulating polymer nanocomposites. ACS Appl. Mater. Interfaces.

[69] Park H., Kim S.E. (2019). Two-step plasma treatment on copper surface for low-temperature Cu thermo-compression bonding. IEEE Trans. Compon. Packag. Manuf. Technol..

[70] Park H., Seo H., Kim S.E. (2020). Anti-oxidant copper layer by remote mode N_2_ plasma for low temperature copper–copper bonding. Sci. Rep..

[71] Park H., Kim S.E. (2019). Nitrogen passivation formation on Cu surface by Ar–N_2_ plasma for Cu-to-Cu wafer stacking application. Microsyst. Technol..

[72] Hung T.-H., Liu P.-J., Wang C.-Y., Chung T.-F., Chen K.-N. (2023). A low-cost passivation for low temperature Cu-Cu bonding using PVD-deposited Cu_3_N. IEEE J. Electron Devices Soc..

[73] Hong Z.-J., Liu D., Hu H.-W., Cho C.-I., Weng M.-W., Liu J.-H., Chen K.-N. (2022). Investigation of bonding mechanism for low-temperature CuCu bonding with passivation layer. Appl. Surf. Sci..

[74] Hsu M.-P., Tsai W.-T., Lee O.-H., Chen C.-Y., Kuo T.-Y., Chang H.-H., Chang H.-C., Hong Z.-J., Chen K.-N. Low temperature Cu/SiO_2_ hybrid bonding using area-selective metal passivation (interface metal) technology for 3D IC and advanced packaging. Proceedings of the 2024 IEEE 74th Electronic Components and Technology Conference (ECTC).

[75] Chiu W.-L., Lee O.-H., Chiang C.-W., Chang H.-H. Low temperature wafer-to-wafer hybrid bonding by nanotwinned copper. Proceedings of the 2021 IEEE 71st Electronic Components and Technology Conference (ECTC).

[76] Wenzel L., Rudolph C., Shehzad A., Mukhopadhyay P., Fulford H.J., Junghaehnel M., Panchenko J. Influence of heat treatment on the quality of die-to-wafer hybrid bond interconnects. Proceedings of the 2024 IEEE 74th Electronic Components and Technology Conference (ECTC).

[77] Bonam S., Panigrahi A.K., Kumar C.H., Vanjari S.R.K., Singh S.G. (2019). Interface and reliability analysis of Au-passivated Cu–Cu fine-pitch thermocompression bonding for 3-D IC applications. IEEE Trans. Compon. Packag. Manuf. Technol..

[78] Premachandran C.S., Choi S., Cimino S., Tran-Quinn T., Burrell L., Justison P. Reliability challenges for 2.5D/3D integration: An overview. Proceedings of the 2018 IEEE International Reliability Physics Symposium (IRPS).

[79] Chen K., Jiang D., Kao N., Lai J. (2006). Effects of underfill materials on the reliability of low-K flip-chip packaging. Microelectron. Reliab..

[80] Chung S.-H., Kim J.T., Kim D.H. (2020). Hybrid carbon thermal interface materials for thermoelectric generator devices. Sci. Rep..

[81] Kuo Y.-H., Tran D.-P., Ong J.-J., Tu K., Chen C. (2022). Hybrid Cu-to-Cu bonding with nano-twinned Cu and non-conductive paste. J. Mater. Res. Technol..

[82] Wang Y., Huang Y.-T., Liu Y., Feng S.-P., Huang M. (2022). Thermal instability of nanocrystalline Cu enables Cu-Cu direct bonding in interconnects at low temperature. Scr. Mater..

[83] Park H., Seo H., Kim S.E. (2021). Characteristics of copper nitride nanolayer used in 3D Cu bonding interconnects. Electron. Mater. Lett..

[84] Park H., Seo H., Kim Y., Park S., Kim S.E. (2021). Low-temperature (260 °C) solderless Cu–Cu bonding for fine-pitch 3-D packaging and heterogeneous integration. IEEE Trans. Compon. Packag. Manuf. Technol..

[85] Lu T.-F., Lee P.-Y., Wu Y.S. (2024). Effect of compressive stress on copper bonding quality and bonding mechanisms in advanced packaging. Materials.

[86] Wang X., Han S., Xiao F. (2024). Cu–Cu direct bonding in air by in-situ reduction of copper oxide with glycerol. Appl. Surf. Sci..

[87] Barako M.T., Isaacson S.G., Lian F., Pop E., Dauskardt R.H., Goodson K.E., Tice J. (2017). Dense vertically aligned copper nanowire composites as high performance thermal interface materials. ACS Appl. Mater. Interfaces.

[88] Jiang H., Wang Z., Geng H., Song X., Zeng H., Zhi C. (2017). Highly flexible and self-healable thermal interface material based on boron nitride nanosheets and a dual cross-linked hydrogel. ACS Appl. Mater. Interfaces.

[89] Cui Y., Qin Z., Wu H., Li M., Hu Y. (2021). Flexible thermal interface based on self-assembled boron arsenide for high-performance thermal management. Nat. Commun..

[90] Xu S., Zhang J. (2020). Vertically aligned graphene for thermal interface materials. Small Struct..

[91] Park W., Guo Y., Li X., Hu J., Liu L., Ruan X., Chen Y.P. (2015). High-performance thermal interface material based on few-layer graphene composite. J. Phys. Chem. C.

[92] Loeblein M., Tsang S.H., Pawlik M., Phua E.J.R., Yong H., Zhang X.W., Gan C.L., Teo E.H.T. (2017). High-density 3D-boron nitride and 3D-graphene for high-performance nano–thermal interface material. ACS Nano.

[93] Guo Y., Wang S., Ruan K., Zhang H., Gu J. (2021). Highly thermally conductive carbon nanotubes pillared exfoliated graphite/polyimide composites. npj Flex. Electron..

[94] Kim T.-R., Joo K., Lim B.T., Choi S.-S., Lee B.J., Yoon E., Jeong S.Y., Yim M.J. Enhancements on Underfill Materials’ Thermal Conductivity by Insulation Coating Layer Control of Conductive Particles. Proceedings of the 2018 IEEE 68th Electronic Components and Technology Conference (ECTC).

[95] Hu Y., Chen C., Wen Y., Xue Z., Zhou X., Shi D., Hu G.-H., Xie X. (2021). Novel micro-nano epoxy composites for electronic packaging application: Balance of thermal conductivity and processability. Compos. Sci. Technol..

[96] Lee Sanchez W.A., Huang C.-Y., Chen J.-X., Soong Y.-C., Chan Y.-N., Chiou K.-C., Lee T.-M., Cheng C.-C., Chiu C.-W. (2021). Enhanced thermal conductivity of epoxy composites filled with Al_2_O_3_/boron nitride hybrids for underfill encapsulation materials. Polymers.

[97] Jiang Y., Shi X., Feng Y., Li S., Zhou X., Xie X. (2018). Enhanced thermal conductivity and ideal dielectric properties of epoxy composites containing polymer modified hexagonal boron nitride. Compos. Part A Appl. Sci. Manuf..

[98] Barani Z., Kargar F., Mohammadzadeh A., Naghibi S., Lo C., Rivera B., Balandin A.A. (2020). Multifunctional graphene composites for electromagnetic shielding and thermal management at elevated temperatures. Adv. Electron. Mater..

[99] Zheng Z., Gu X., Liao S.-Y., Ouyang H., Sun R., Zhu P., Wan Y.-J. (2025). Electrically insulating yet excellent EMI shielding FeSiAl/CNF composite film with thermal conductivity for electronic packaging applications. ACS Appl. Mater. Interfaces.

[100] Lee W., Kim J. (2021). Highly thermal conductive and electrical insulating epoxy composites with a three-dimensional filler network by sintering silver nanowires on aluminum nitride surface. Polymers.

